# Integrated device for optoacoustically-guided ultrasound surgery

**DOI:** 10.1038/s41467-026-75692-4

**Published:** 2026-07-21

**Authors:** Isaac Esteban, Nima Mahkam, Yi Chen, Martha Gjikolaj, Beat Werner, Henning Richter, Katrin Beckmann, Edin Nevzati, Daniel Coluccia, Hikari A. I. Yoshihara, Frauke Seehusen, Daniel Razansky, Hector Estrada

**Affiliations:** 1 Institute for Biomedical Engineering, University of Zurich and ETH Zurich, Zurich, Switzerland; 2 Institute of Pharmacology and Toxicology, University of Zurich, Zurich, Switzerland; 3 Brain Research Institute, University of Zurich, Zurich, Switzerland; 4 Center for MR-Research, University Children’s Hospital Zurich, Zurich, Switzerland; 5 Diagnostic Imaging Research Unit (DIRU), Clinic for Diagnostic Imaging, Vetsuisse Faculty, University of Zurich, Zurich, Switzerland; 6 Faculty of Medicine, University of Basel, Basel, Switzerland; 7 Department of Neurosurgery, University of Colorado Anschutz Medical Campus School of Medicine, Aurora, CO USA; 8 Neurosurgery, Stadtspital Zurich, Zurich, Switzerland; 9 Laboratory for Animal Model Pathology (LAMP), Institute of Veterinary Pathology, Vetsuisse Faculty, University of Zurich, Zurich, Switzerland

**Keywords:** Biomedical engineering, Translational research

## Abstract

High-intensity focused ultrasound has emerged as a promising tool for treating tumors and other pathologies by inducing localized thermal lesions. However, treatment outcomes depend strongly on accurate coverage of the treated region and monitoring lesion formation in real time. While magnetic resonance imaging has successfully been used to guide high-intensity focused ultrasound therapy, it significantly increases the cost and complexity of the treatment. To address these limitations, we have developed a system that integrates real-time three-dimensional tomographic optoacoustic imaging with concurrent focused ultrasound emission using a high-frequency spherical array transducer. We evaluated the performance of the system in the mouse brain ex vivo and further assessed its clinical translatability in a living sheep, demonstrating successful induction of a confined thermal lesion beneath the cortical surface, as confirmed by magnetic resonance imaging and histology. The system enabled precise treatment targeting, while optoacoustic signal changes during sonication provided a reliable indicator of thermal lesion progression. This capability allowed on-the-fly adjustment of the thermal dose, helping to minimize the risk of vascular damage or adverse effects on off-target structures.

## Abstract (web summary)

High-intensity focused ultrasound can treat disease by heating tissue, but monitoring the treatment is difficult. Here, the authors use 3D optoacoustic imaging integrated with ultrasound therapy to safely guide the treatment in animal models, paving the way for safer, more precise brain therapies.

## Introduction

High-intensity focused ultrasound (HIFU) is a noninvasive therapeutic modality that concentrates acoustic energy to ablate tumors or other pathological tissues. By inducing localized heating, HIFU leads to coagulative necrosis while sparing surrounding healthy structures^1,2^. To achieve this, ultrasonic waves are focused using a concave transducer or phased array to target specific tissue regions with high precision^3,4^. This concentration of energy generates a localized temperature increase sufficient to induce protein denaturation, irreversible cellular damage, and ultimately, tissue ablation^5^. While flat phased arrays offer advantages, such as more flexible focal point adjustment, concave arrays are more efficient in concentrating energy at the target site, making them the design of choice for HIFU applications^6^.

HIFU can be monitored effectively using invasive methods, such as thermocouples or fiber-optic temperature sensors^7^. However, these methods provide temperature readings from a limited number of discrete points. In contrast, noninvasive imaging is the most versatile approach to assess the extent of the treated region. Magnetic resonance image-guided high-intensity focused ultrasound (MRgHIFU) combines magnetic resonance imaging (MRI) with quantitative MR thermometry, enabling highly sensitive detection of tissue temperature changes and real-time monitoring of HIFU ablation^8–10^. However, its high cost, the need for MRI-compatible materials, and prolonged procedure times limit its widespread clinical use. Ultrasound-imaging-guided HIFU (USgHIFU), featuring significantly lower hardware costs, can provide real-time monitoring with high spatial resolution as well as sizable penetration depth >10 cm^11^. While temperature variations affect the speed of sound in the treated region, reflected US signals manifest low sensitivity to temperature changes, and thus pulse-echo US imaging is not capable of accurate HIFU guidance^12–14^.

Optoacoustic (OA) tomography is a noninvasive imaging modality that detects ultrasound waves generated by light-absorbing molecules upon pulsed laser excitation^15–18^. OA imaging is particularly well-suited for vascularized tissues, where endogenous hemoglobin serves as a strong optical absorber, providing intrinsic contrast^19–21^. Notably, OA signals exhibit strong temperature dependence^22,23^, allowing for the monitoring of tissue heating. Below 50 °C, relative changes in temperature can be quantified using the linear dependence of temperature with the Grüneisen parameter^24^. At higher temperatures, however, cellular damage and protein denaturation permanently alter tissue optical properties, breaking this linearity and diminishing temperature quantification accuracy^25,26^.

OA imaging has been used to monitor tissue coagulation induced by various thermal therapies^27–31^. OA-guided systems have also been proposed as a means to monitor HIFU procedures^32,33^. Such approaches are particularly suited for this purpose due to intrinsic sensitivity to temperature-induced changes in tissue and the ability to detect alterations in tissue composition resulting from thermal effects^34–36^. Prior studies have demonstrated the feasibility of using OA signal amplitude as an indirect measure of temperature during HIFU treatments, based on the correlation between signal intensity and tissue temperature^4,32^. However, this relationship is influenced by multiple tissue-specific factors, thus restricting estimations to temperatures below 50 °C and requiring prior calibration for accurate temperature estimation. Other studies have leveraged the increased optical absorption and decreased scattering coefficient in heated tissue to detect HIFU-induced thermal changes^6,34,37^. Despite their potential, integrating optical systems with the bulky opaque transducers needed for imaging and HIFU delivery remains technically challenging, reducing their suitability in real surgical scenarios. As a result, previously reported optoacoustically-guided HIFU systems have employed different transducers for imaging and therapy^32,38^. This configuration requires frequent and time-consuming coregistration to maintain alignment and prevent damage to adjacent healthy tissues, thereby impeding dynamic steering under real-time imaging guidance and increasing the complexity and variability of the procedure.

Here, we present an integrated optoacoustically guided HIFU system (OAgHIFU) that combines 3D real-time OA tomography with concurrent steerable focused ultrasound (FUS) delivery for inducing thermal lesions. Since the same spherically focused array is used for both purposes, the interleaved sonication and imaging are seamlessly coregistered via the acoustic time-reversal principle. This unified design simplifies operation and enhances both procedural efficiency and system reliability. The system’s performance is demonstrated in vivo in a large-animal model, underscoring its potential for translational image-guided ultrasound therapies.

## Results

### OA-guided HIFU system

**Fig. 1:**
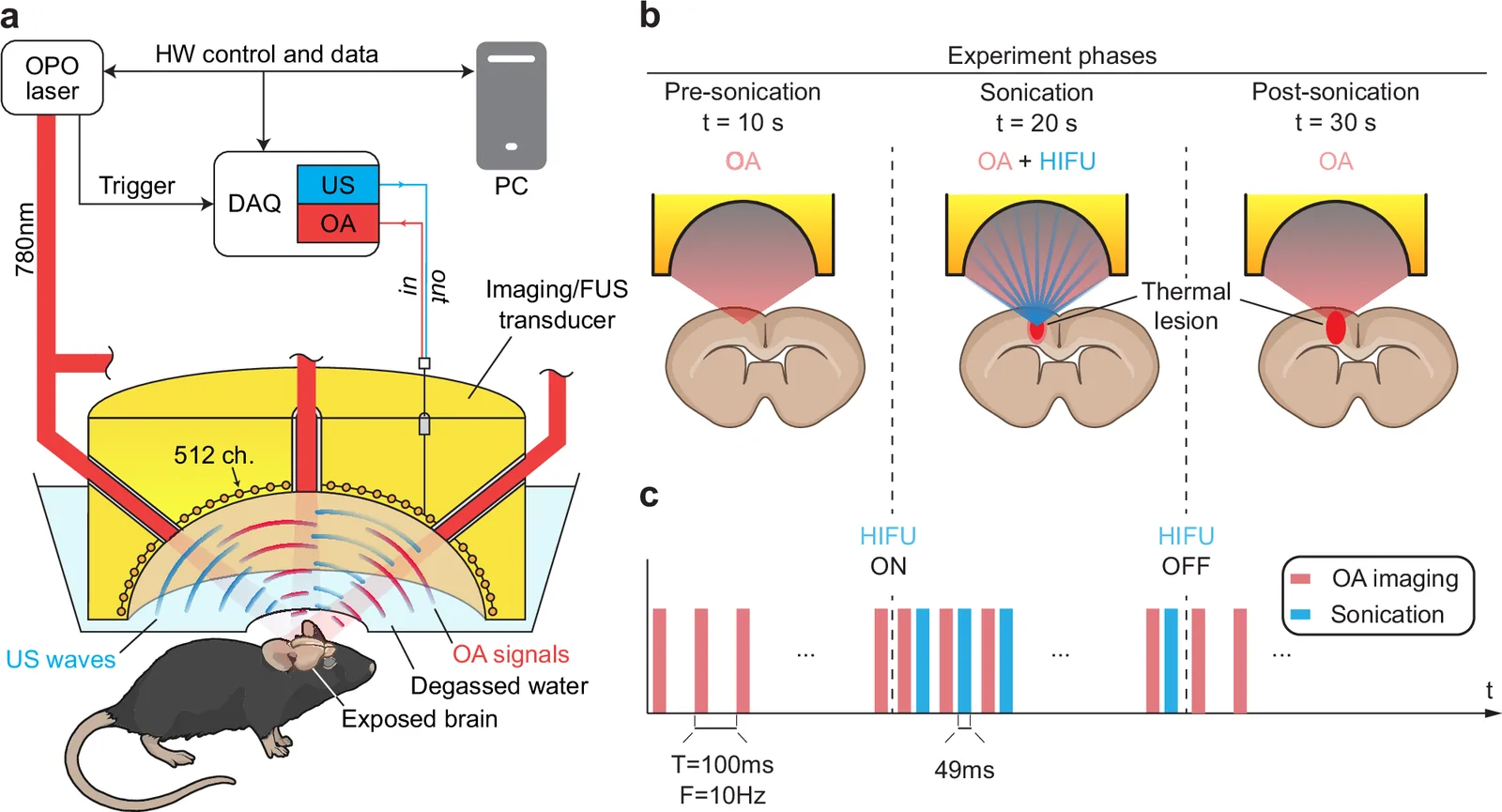
Optoacoustic guided HIFU (OAgHIFU) system and experiment timeline. **a** Schematics of the experimental setup, including electronic components and the animal model. The curve-shaped transducer is immersed in a degassed water bath. The personal computer (PC) is used for configuring the laser and the acquisition system for both imaging and high-intensity focused ultrasound (HIFU) emission. The data acquisition electronics (DAQ) outputs ultrasound (US) waves and reads optoacoustic (OA) signals. HW, hardware. Mouse illustration adapted from NIAID NIH BioArt Source (bioart.niaid.nih.gov/bioart/279). **b** Experiments are divided into three phases: OA baseline acquisition for 10 s, OA imaging with interleaved HIFU emission for 20 s, and final OA image acquisition for 30 s. During sonication, the tissue undergoes a thermal lesion due to progressive heating of the focal region. Coronal section illustration from NIAID NIH BioArt Source (bioart.niaid.nih.gov/bioart/370). **c** During the experiment, the system is continuously recording OA data. The optical parametric oscillator (OPO) laser triggers the acquisition system at 10 Hz. During sonication, HIFU emissions are interleaved with OA at a rate of 10 Hz, and emitting HIFU during 49 ms.

**Fig. 2:**
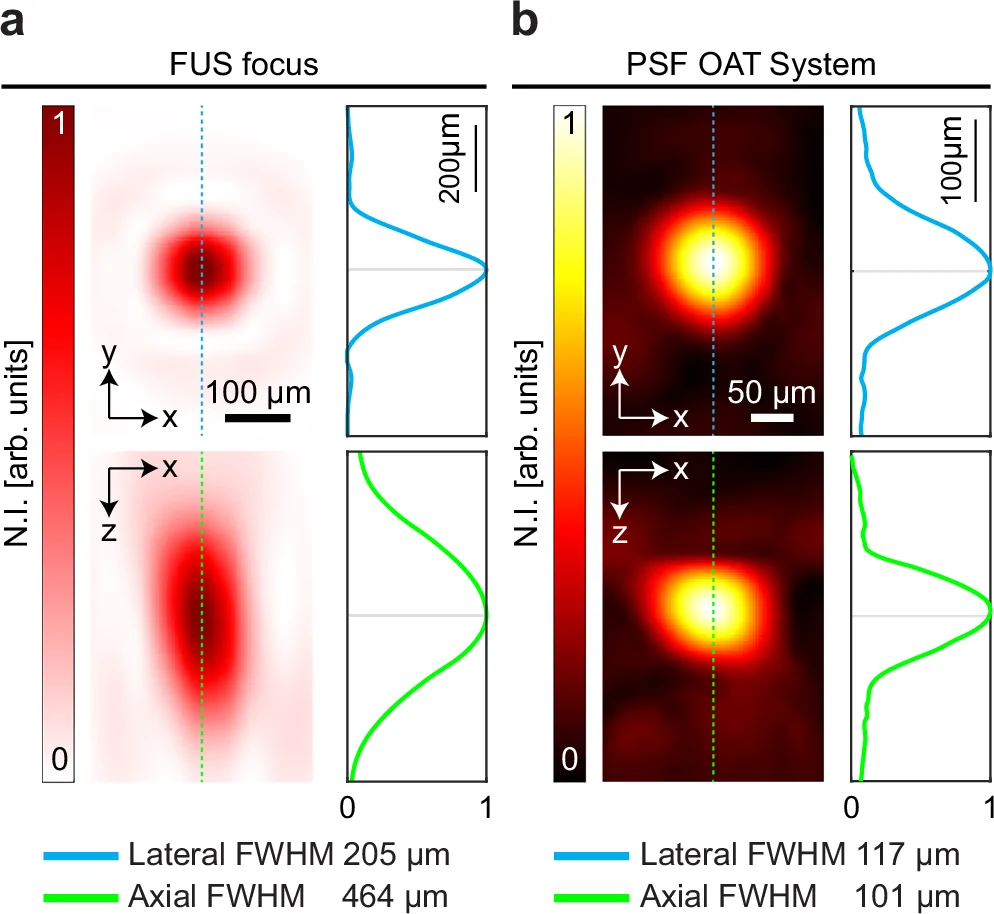
FUS focal size and OA spatial resolution. **a** Focused ultrasound (FUS) transducer focal region scans at three orthogonal planes for a 5 MHz emission. Maximum intensity projections (MIPs) of the normalized pressure maps reveal an ellipsoidal focus, elongated in the axial direction. Lateral (blue) and axial (green) beam width profiles along the dashed lines are represented. Full width at half maximum (FWHM) indicated by labels. **b** Point spread function (PSF) of the OA tomography (OAT) system. MIPs of the OA volumetric image of a ~30 µm diameter polyethylene microsphere. Lateral (blue) and axial (green) OA MIP image profiles along the dashed lines are represented. FWHM indicated by labels.

The OAgHIFU system (Fig. 1a) enabled real-time optoacoustic imaging with a laser operating at 780 nm wavelength for anatomical navigation and target localization in the ex vivo mouse brain (Supplementary Fig. 1a), following skull removal and sample positioning. The experimental protocol consisted of three consecutive phases (Fig. 1b). First, a 10 s baseline of OA imaging was acquired prior to sonication (Fig. 1c) and then used to compute the relative OA signal change (ΔOA/OA). Second, a 20 s period of interleaved ultrasound (US) sonication and OA imaging was performed. Finally, OA imaging continued for an additional 30 s post-sonication. The spatiotemporal resolution of both the focused US field (Fig. 2a) and the OA imaging (Fig. 2b) enabled precise monitoring and localized treatment of the target region.

### OA monitoring of HIFU-induced lesions

**Fig. 3:**
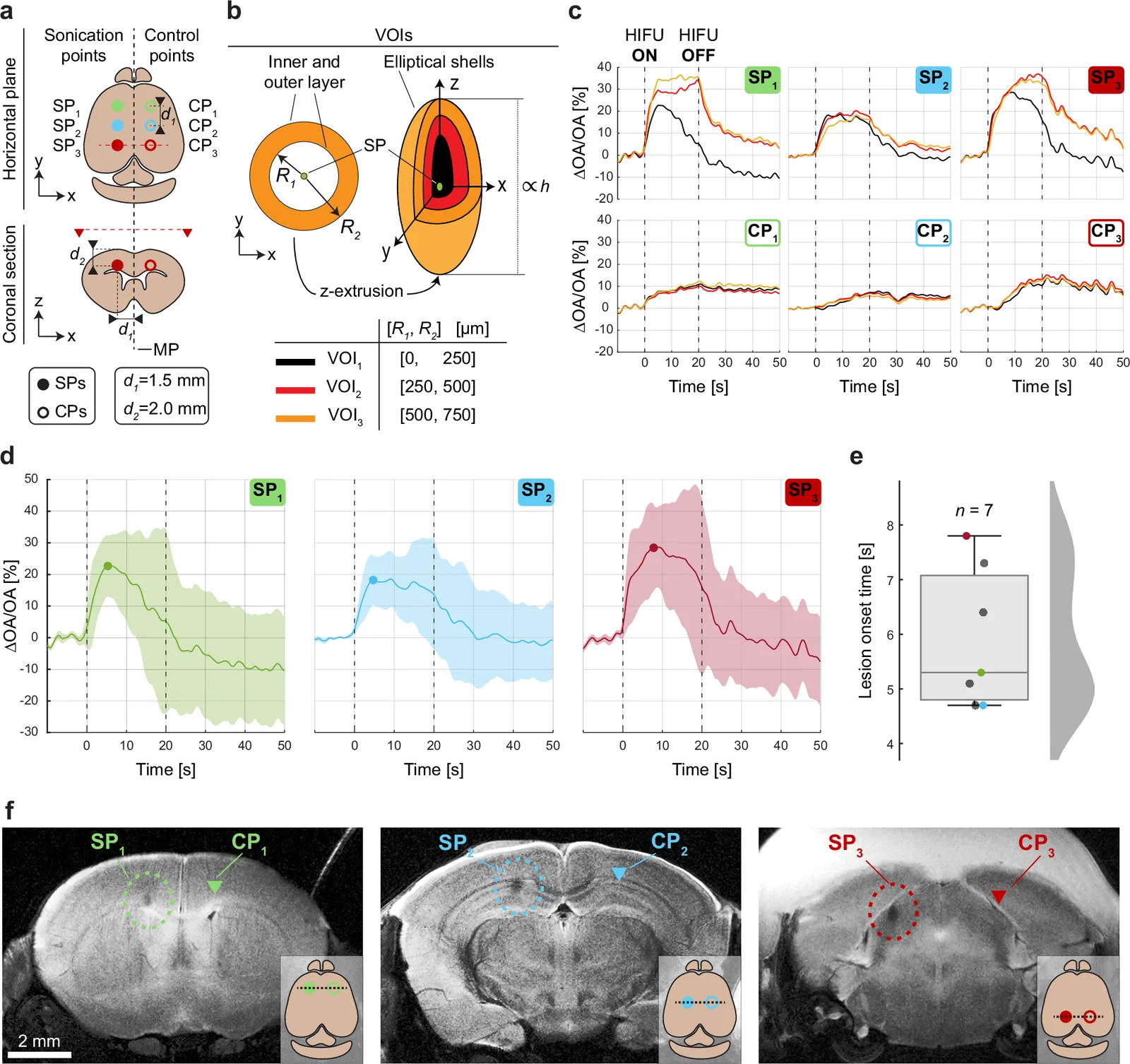
OA traces suggest the onset of tissue thermal damage. **a** Different FUS foci (sonication points, SPs) are distributed in the left hemisphere along the anterior-posterior axis, separated 1.5 mm (*d*_1_) from each other and from the midplane (MP); 2 mm below the surface (*d*_2_). Three points in the right hemisphere are used as control points (CPs). **b** Volumes of interest (VOIs) are defined with an inner and outer radius (*R*_1_ and *R*_2_, respectively) contained within the brain horizontal plane. The planar circular shell is extruded along the z-axis, considering the factor *h* (“Methods”). **c** For all the SPs and CPs, ΔOA/OA traces are averaged across all the voxels within their respective VOI, indicated by color labels in (**b**). **d** Mean ΔOA/OA traces for VOI_1_ at the SPs shown in (**a**). Solid lines indicate the mean ΔOA/OA across all voxels within VOI_1_ (*n* = 257 voxels), and shaded regions indicate ±1 s.d. across voxels at each time point. Dots indicate the estimated lesion onset time. **e** Boxplot and violin plot of the estimated lesion onset times at the SPs, all obtained from three different mice. Individual dots represent independent sonication points (*n* = 7): colored dots correspond to the SPs shown in (**a**), and gray dots correspond to additional SPs. The violin plot shows the kernel density distribution. In the box plot, the center line indicates the median, the box limits indicate the first and third quartiles, and whiskers indicate the minimum and maximum values. **f** T2 MRI coronal scans performed after sonication reveal regions with different contrast, comparing SPs regions (left hemisphere, delineated by dotted-line contours) to CPs regions (right hemisphere, pointed by solid colored arrows). Image contrast is enhanced to highlight the dark region in the SPs.

We selected three sonication points (SPs) in the left hemisphere (SP_1_, SP_2_, and SP_3_) spaced 1.5 mm apart along the anterior-posterior axis, positioned 1.5 mm lateral from the midplane, and 2 mm below the brain surface (Fig. 3a). We calculated the average ΔOA/OA of all the voxels embedded within volumes of interest (VOIs), shaped as concentric ellipsoidal shells surrounding the focus (Fig. 3b top row, “Methods”).

In the initial seconds of the sonication, the ΔOA/OA traces from all SPs show an expected increase in OA amplitude driven by the temperature rise due to ultrasonic heating^ 25^. Thereafter, the innermost VOI (black curves, Fig. 3c) quickly changes to a downward trend, while the two external VOIs (red and orange lines in Fig. 3c) do not exhibit a similarly pronounced downward trend but instead plateau until sonication finishes. At the control points (CPs), the OA signal does not change significantly (Fig. 3c, bottom row) as they are only minimally affected by heat diffusing from the focal region.

The onset of thermal lesion formation is most likely indicated by the time point at which the initial upward trend in OA signal reverses, reflecting permanent changes in the tissue’s optical properties induced by thermal damage (Fig. 3d). To quantify this, we analyzed the VOI closest to the acoustic focus (3 mice, 7 SPs) and determined a consistent lesion onset time of approximately 5 s (Fig. 3e).

Following HIFU treatment, we imaged the mouse head in a 7 T MRI scanner to assess tissue alterations (“Methods”). This preliminary assessment revealed hypointense areas at the SPs (dotted-line contours in Fig. 3f), with respect to the CPs (solid colored arrows in Fig. 3f) in T2-weighted images. The reduced signal intensity in the SPs is consistent with tissue alterations induced by focused ultrasound, potentially leading to a local decrease in T2 relaxation times. This effect may be attributed to protein denaturation or early coagulative processes, as commonly observed in thermally damaged tissue^39,40^.

To better visualize the FUS-induced tissue response, we registered the 3D volumetric OA data to the T2-weighted MRI images (Supplementary Fig. 1b and “Methods”). This co-registration enabled anatomical visualization of ΔOA/OA at the sonication sites over time (Supplementary Fig. 2a), as well as a phase-wise comparison of the normalized ΔOA/OA response across all the SPs (Supplementary Fig. 2b and Supplementary Movie 1).

**Fig. 4:**
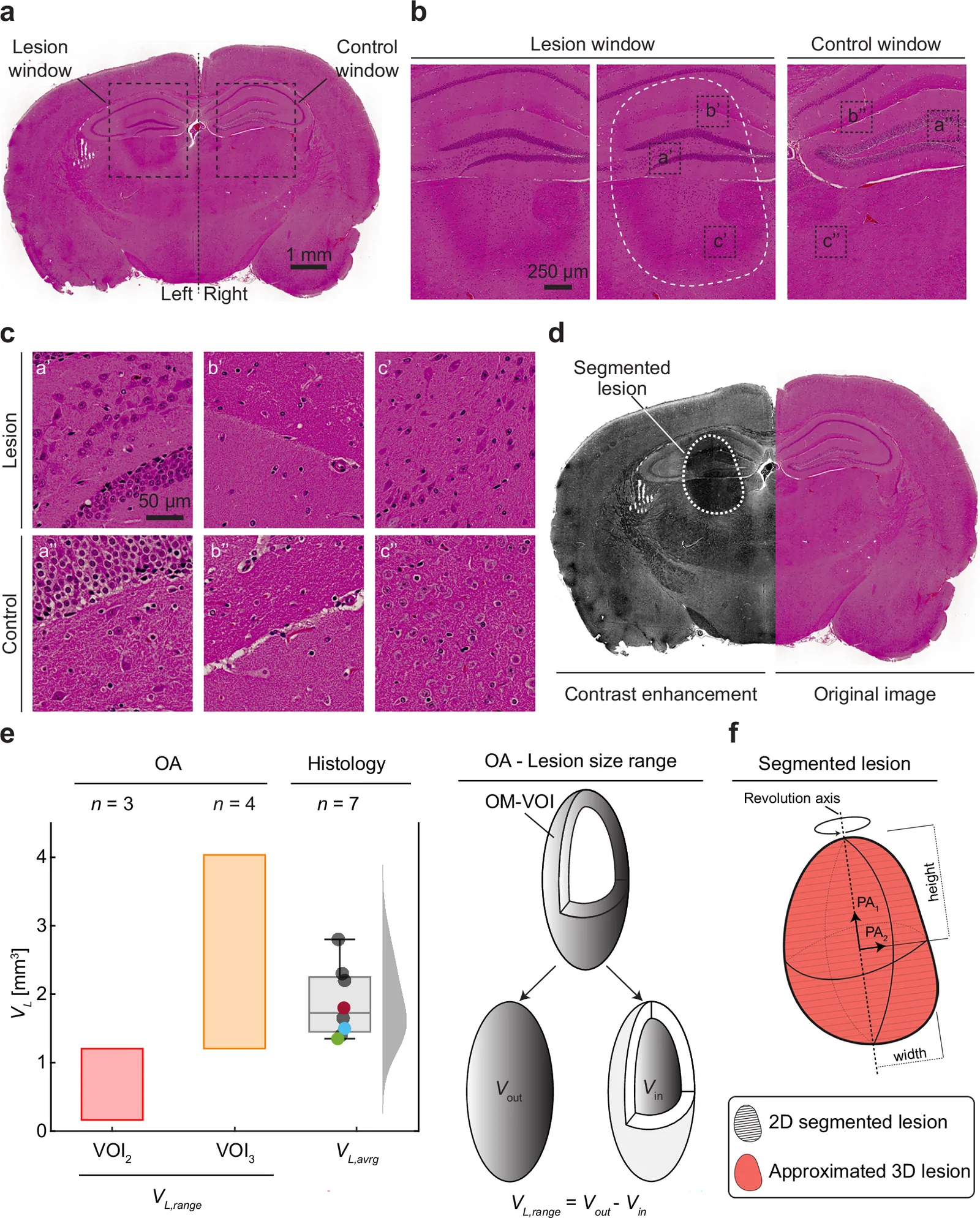
Tissue lesion induced by FUS is revealed by hematoxylin and eosin histology. **a** Coronal section representing the region targeted by SP_1_ (Fig. 3a). The left side shows a dotted-line box around the FUS region (lesion window), as well as the right side, acting as control (control window). **b** Representative H&E-stained images of the lesion and contralateral control windows. In the lesion window, an ellipsoidally shaped darker region indicates the lesion extent; the white dashed contour highlights the segmented lesion region. Dashed boxes indicate ROIs shown at higher magnification in (**c**). **c** Higher-magnification views of the selected ROIs from the lesion and control windows. Similar lesion-associated histological features were observed across *n* = 7 independent thermal lesions from three mice. **d** Contrast-enhanced histological section highlights the extent of the lesion. The white contour represents the outer boundary of the segmented lesion. **e** Optoacoustic (OA)-based lesion-size estimation and comparison with histology-derived lesion volumes. OA analysis assigns each lesion to a volume range (*V*_*L*, *range*_) defined by the inner and outer volumes of the outermost volume of interest (OM-VOI) classified as lesioned (“Methods”). Red and orange range bars indicate the *V*_*L*, *range*_ corresponding to VOI_2_ (*n* = 3 lesions) and VOI_3_ (*n* = 4 lesions), respectively. Histology-derived lesion volumes (*V*_*L*_) were obtained by segmentation of H&E-stained sections from *n* = 7 thermal lesions and are shown as individual dots with a boxplot and violin plot. Colored dots correspond to SP_1_, SP_2_, and SP_3_ shown in Fig. 3a; gray dots correspond to additional lesions. The violin plot shows the kernel density distribution. In the boxplot, the center line indicates the median, box limits indicate the first and third quartiles, and whiskers indicate the minimum and maximum values. **f** The principal axes (PA) of the segmented lesion allow estimating height and width. The 2D segmented lesion is revolved around the primary principal axis (PA_1_), serving as the axis of revolution. The resulting solid of revolution provides an approximation of the lesion volume.

To further assess the effects of FUS exposure, we performed histological analysis on tissue sections from all the SPs (“Methods”). For each brain, hematoxylin and eosin H&E-stained sections included both the lesion and the contralateral control region (Fig. 4a). Notably, the lesion region exhibits a darker color than the control (Fig. 4b, white dashed contour). Closer inspection of different ROIs at the lesion window (Fig. 4c, top row) and the control window (Fig. 4c, bottom row) showed differences in cytoplasmic water content, suggesting a process of cell dehydration caused by the temperature rise during FUS (Discussion). To facilitate clearer differentiation between the affected and control regions, we applied an objective contrast enhancement method based on the quantifiable difference in staining intensity associated with cytoplasmic water content, thereby improving the delineation of lesion boundaries (Fig. 4d and “Methods”).

Next, we used the OA signals to estimate dimensions of the induced lesions (“Methods”). Specifically, we identified the outermost VOI (OM-VOI) that encapsulates the lesion, and extracted the lesion size range (*V*_*L*, *range*_, Fig. 4e). Based on this analysis, 3 out of 7 SPs were assigned to the *V*_*L*, *range*_ corresponding to VOI_2_, including SP_2_ and SP_3_ (Fig. 4e, red range plot and Supplementary Fig. 3) whereas the remaining 4 to the *V*_*L, range*_ corresponding to VOI_3_, including SP_1_ (Fig. 4e, orange range plot and Supplementary Fig. 3). To evaluate these estimates, we calculated the lesion volume (*V*_*L*_) in the H&E-stained sections as ground truth, using the enhanced-contrast images (Fig. 4d), obtaining an average lesion size *V*_*L*, *avrg*_ = 1.72 mm^3^ (*n* = 7, Fig. 4e, f). In addition, to assess the correspondence between the OA-based range estimate and histology on a lesion-by-lesion basis, we compared the OA-estimated volume range with the histology-derived lesion volume for each of the seven lesions (Supplementary Fig. 3). This comparison showed direct agreement in 4 of the 7 lesions. In the remaining 3 lesions, the OA-based estimate fell in the adjacent lower volume range while remaining close to the corresponding histology-derived volume, indicating conservative underestimation rather than substantial discrepancy. Using the midpoint of each OA-estimated lesion-volume range as a simple summary statistic, the comparison with histology yielded a mean discrepancy of −0.17 mm^3^ and a mean absolute discrepancy of 0.73 mm^3^, indicating limited overall bias and moderate lesion-by-lesion deviations (“Methods”). Together, these results support the use of the OA-based framework as a bounded, range-based estimate of lesion size rather than precise volumetric quantification.

### In vivo validation in a large animal model

**Fig. 5:**
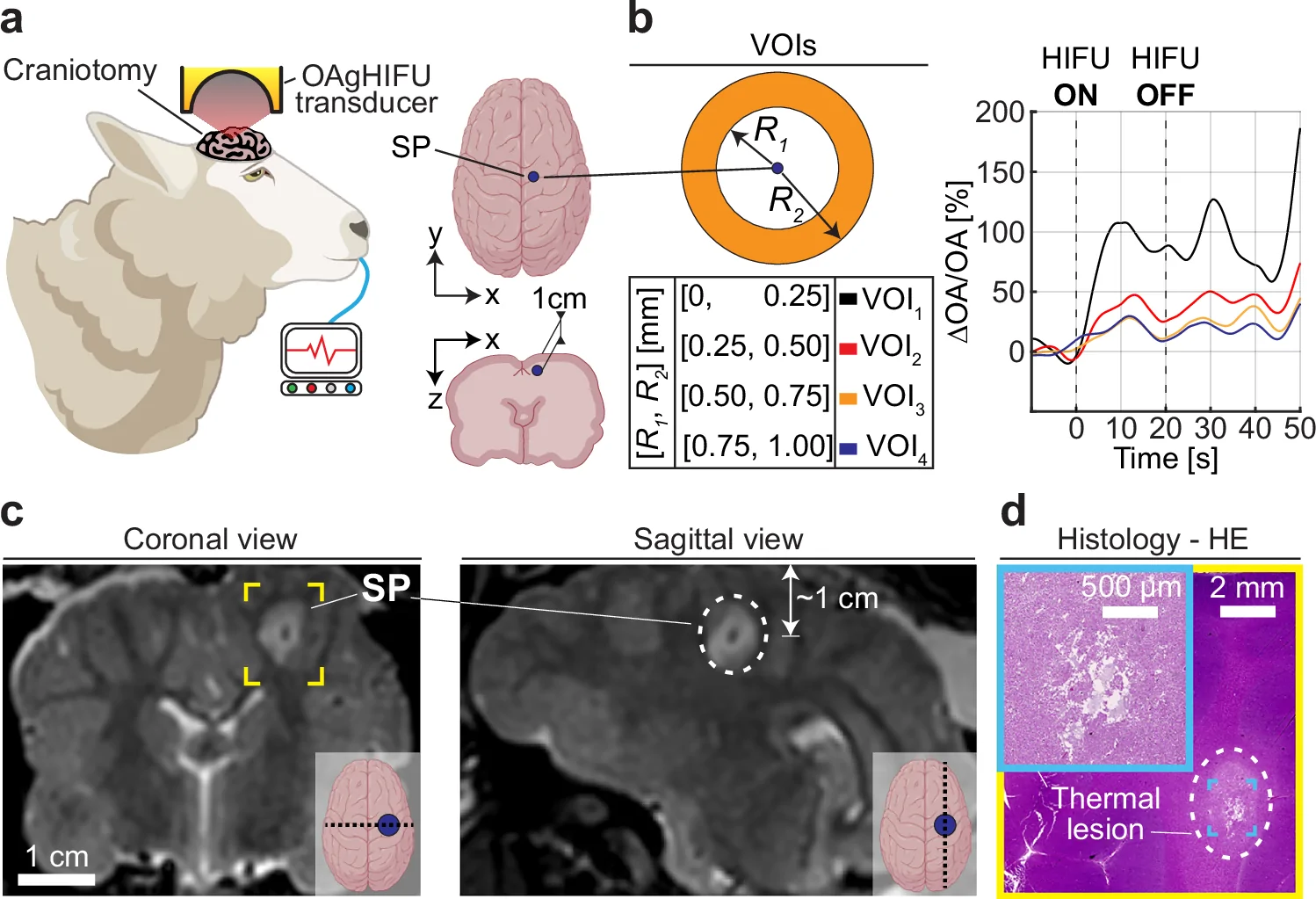
Setup, monitoring, and post-therapy assessment of OAgHIFU in an in vivo sheep model. **a** Within the clinical environment, the in vivo sheep is anesthetized, and the optoacoustically guided HIFU (OAgHIFU) transducer is positioned directly atop the brain through the craniotomy opening. The sonication point (SP) was located on the right hemisphere, close to the midplane, placed 1 cm below the cortical surface. **b** For each volume of interest (VOI), the mean ΔOA/OA trace is computed by averaging the signal across all constituent voxels. The VOIs are centered on the SP and are color-coded as shown. **c** Post-therapy in vivo T2-weighted coronal MRI revealed hyperintense regions with central hypointensity at the SP. The region outlined (yellow) is enlarged in (**d**). H&E-stained histological section of the sonicated region from the in vivo sheep experiment. The yellow-framed image corresponds to the region outlined in yellow in (**c**). The white dashed contour indicates the thermal lesion, and the cyan dashed box indicates the region shown at higher magnification in the cyan-framed inset. This large-animal in vivo experiment was performed once (*n* = 1 animal) and was not independently repeated. The sheep illustration and top-view brain schematic in (**a**, **c**) were created in BioRender. Mahkam, N. (2026) https://BioRender.com/tciyd5c and adapted by the authors using Adobe Illustrator 2026.

We conducted in vivo validation in a craniotomized sheep model under anesthesia to evaluate the intraoperative translational potential of OAgHIFU (Fig. 5a, left). The transducer was adapted for a larger-scale brain while keeping all other setup elements and parameters unchanged. Specifically, elements with a lower central frequency were used to increase the imaging penetration depth (“Methods”). The OAgHIFU system further enabled real-time OA imaging with a laser operating at a wavelength of 780 nm for anatomical navigation and target localization in the sheep brain (Supplementary Fig. 1c).

We defined a SP 1 cm below the cortical surface (Fig. 5a, right), located in the mesial temporal-diencephalic junction, involving adjacent hippocampal, thalamic/hypothalamic, and piriform lobe structures. We computed the mean ΔOA/OA for the different VOIs, using larger values of *R*_1_ and *R*_2_ to account for the larger focus (Figs. 3b, 5b, and “Methods”). In the resulting traces, ΔOA/OA for all VOIs starts to increase because of the temperature rise from the FUS-mediated thermal energy deposition. Approximately 9 s into sonication, VOI_1_ undergoes a marked trend change, followed by VOI_2_ approximately 3 s after. VOI_3_ and VOI_4_ show minor corrections. These results indicate that the lesion-associated OA response is clearly captured within VOI_2_. Since the in vivo traces exhibited motion-related and physiological fluctuations, we refined the lesion size by examining the temporal derivative of ΔOA/OA during sonication (“Methods” and Supplementary Fig. 4). This refined analysis localized the lesion boundary within VOI_3_, corresponding to an estimated lesion width of 1–1.5 mm.

After HIFU, we performed a postoperative MRI (Fig. 5c and “Methods”). The assessment of T2-weighted MRI reveals a clearly distinguishable area in the target SP, characterized by a hyperintense area with central hypointensity. The lesioned area was evaluated in formalin-fixed paraffin-embedded brain tissue sections. In the H&E-stained sections, rarefication of the cerebral parenchyma was observed in the white matter of the cerebrum. In addition to tissue ruptures, small accumulations of pale eosinophilic fluid were present. Increased cellularity in terms of gliosis or inflammatory cell infiltration was not detected (Fig. 5d and “Methods”). In the corresponding histological section, the lesion measured approximately 1.6 mm in width, which is close to the refined OA-based estimate range, noting that the histological assessment was based on a single 2D section and therefore provides a partial representation of the full 3D lesion extent.

## Discussion

We have demonstrated the functionality of our integrated device, capable of real-time OA imaging concurrently with FUS sonication, and proven its performance in an in vivo animal model. The system utilizes a spherically focused transducer array with a larger aperture angle, enabling single-pulse acquisition of volumetric OA images. Operating at 780 nm wavelength in the near-infrared (NIR) window, the system achieves high-resolution imaging of brain vasculature with greater penetration depth as compared to shorter wavelengths. At this wavelength, reduced light scattering and absorption in tissues help preserve the essential spectral features of hemoglobin, thus enhancing vascular contrast^41^. Using real-time OA imaging, we successfully navigated the brains of different animal models and defined target volumes for FUS application. During sonication, we monitored average ΔOA/OA within different VOIs to detect the onset of thermal lesions. Consistent OA signal changes were observed throughout the sonication phase, demonstrating the system’s capability for targeted heating with simultaneous volumetric imaging. Notably, ΔOA/OA provided a reliable real-time biomarker for thermal lesion formation, without relying on actual temperature measurements, as independently validated by MRI and histological analysis.

In heat-induced tissue lesions, several factors related to optical absorption and scattering may vary with temperature, and thus need to be considered. Optical absorption depends on intrinsic tissue properties, which are contributed by various chromophores. Below 50 °C, significant thermal denaturation and structural changes do not occur in most tissues; therefore, absorption remains constant while optical scattering experiences minimal changes^42,43^. Above 50 °C, proteins like collagen begin to denature, causing significant changes in optical absorbance^44^. Structural changes in cells lead to a mismatch in the refractive index between tissues, increasing scattering and reducing optical fluence^45,46^. Ultimately, this leads to a non-linear Grüneisen coefficient and temperature dependence due to irreversible changes in the optical properties of the tissue. These aspects leave an imprint in the time-lapse OA signal by causing a drastic change of tendency, which was consistent across multiple ablation points, in different specimens. Such behavior may serve as an indication of the onset of a thermal ablation. We attribute the differences in the ΔOA/OA traces to the heterogeneity in tissue properties across the various brain regions targeted as the focal locations, which also influences the lesion onset time.

We observed a distinctive ΔOA/OA profile in some of the traces (e.g., SP_1_, Fig. 3c) where the OA signal increase in the outer VOIs surpassed that of VOI_1_. This pattern, which would be paradoxical if the OA signal were a simple function of temperature, is consistent with a hypothesis of dynamic optical property change during coagulation. As the focal core coagulates, its optical scattering increases irreversibly^34^. This depletes the local optical fluence available for OA conversion. Simultaneously, light scattered from this opaque region may enhance the fluence (and thus the OA signal) in the surrounding heated tissue. Consequently, the spatial peak of the OA signal can transiently mark the advancing coagulation boundary rather than the temperature maximum. We posit that this phenomenon is mediated by local optical heterogeneity and the specific spatiotemporal progression of coagulation, explaining why it is prominently observed only in a subset of the acquired datasets.

MRI is a robust technique for assessing brain tissue water content, making it well-suited for detecting changes associated with thermal damage following sonication, such as protein denaturation and T2 relaxation time shortening. The observed reduction in signal intensity within the SPs is consistent with these ultrasound-induced tissue alterations and likely reflects localized decreases in T2 relaxation times. Such changes are indicative of early thermal effects, including protein denaturation and the onset of coagulative processes, which are well-documented markers of thermally damaged tissue^39,40^.

Using the MRI scans, we were able to identify tissue alterations in regions corresponding to the SPs, seen as hypointense areas. In the mouse model, the strength of this reproducibility is further supported by MRI from multiple specimens, each sonicated in different regions along the anterior-posterior axis, all of which showed consistent lesion size and depth. These findings align with previous reports where FUS-treated regions were similarly presented as hypointense zones on T2-weighted MRI scans^47^. However, in the sheep model, the hypointense alteration is enveloped by a hyperintense tissue. The latter may be due to vacuolization surrounding the lesion, probably caused by edema. Additionally, registering OA and T2 MRI data allowed us to visualize the temporal dynamics of the ablated regions within a spatial reference. Finally, we performed a histological study using formalin-fixed and paraffin-embedded tissue with subsequent H&E staining conducted according to standard protocols. In the ex vivo mouse brain samples, we found differences in cellular structures in the FUS-targeted region, which are based on cellular water content. In the target regions, the histology shows nuclei embedded within cytoplasmic regions, characterized by low eosin affinity, and therefore appear as white areas in the images. This reduced affinity is attributable to the high water content of cells within their cytoplasm. However, the lesion region is more eosinophilic, i.e., appears more pink/red. As FUS is being delivered, the heat leads to cellular dehydration, which in turn contributes to the condensation and aggregation of cytoplasmic proteins^ 48^. The denaturation caused by the heating exposes more binding sites for the eosin dye. On top of that, as the cytoplasm loses water, it shrinks and becomes more condensed, leading to the appearance of reduced cytoplasmic volume around the nucleus^49^. This dehydration contributes to the destruction of cell membranes and intracellular structures^50^. Regarding the in vivo sheep brain histology, the H&E-stained sections revealed mechanical disruption of the white matter, characterized by parenchymal loosening and tissue rupture, in the absence of a discernible gliotic or inflammatory response.

To enable practical lesion size estimation in real time, we introduced a range-based approach (*V*_*L*, *range*_) derived from OA signals. This method allowed us to consistently estimate the lesion extent across SPs. To validate this approach, we compared the OA-based estimates with histological measurements obtained from contrast-enhanced H&E sections, which yielded an average lesion volume of *V*_*L*, *avrg*_ = 1.72 mm^3^. A lesion-by-lesion comparison further showed that the OA-based framework provides a bounded estimate of lesion extent in most cases, while the remaining mismatches reflected conservative underestimation of lesion size (Supplementary Fig. 3). Consistent with this interpretation, midpoint-based comparison yielded a mean discrepancy of −0.17 mm^3^ and a mean absolute discrepancy of 0.73 mm^3^, indicating limited overall bias despite the deliberately coarse range-based formulation.

In the in vivo sheep experiment, a refined derivative-based analysis yielded an estimated lesion width of 1–1.5 mm, which aligns closely with the histological measurement of approximately 1.6 mm (Fig. 5d). These findings validate the method’s efficacy for real-time monitoring while simultaneously highlighting its limitations in achieving exact volumetric delineation.

In this context, the current lesion-evaluation framework should be interpreted as an empirical range-based method for intra-procedural assessment. Our implementation infers the lesion extent from discrete concentric ellipsoidal VOIs centered on the acoustic focus, such that the resulting estimate is bounded by the shell spacing used in the analysis. Its precision is further influenced by the spatial resolution of the OA system, the finite voxel size, the elongated geometry of the HIFU focus, and tissue-dependent thermal and optical heterogeneity. While comparisons with histology are further complicated by uncertainties in tissue sectioning and co-registration, the approach remains highly relevant; it provides critical real-time feedback by detecting lesion onset and ensuring the ablated region remains within the intended treatment zone. Future work should focus on improving lesion delineation through continuous 3D boundary estimation from the OA signal distribution, quantification strategies that explicitly account for the system point spread function (PSF), or combining OA monitoring with complementary imaging modalities, such as pulse-echo ultrasound for improved boundary definition and validation.

Aiming for an in vivo application of our integrated device, we have tackled several key aspects, such as the potential for blood vessel rupture and the influence of nearby vasculature on tissue heating. Preventing vessel rupture is important to avoid internal hemorrhages and other complications^51,52^. On the other hand, blood vessels act as heat sinks, dissipating thermal energy and leading to uneven thermal distribution, which in turn affects the size and shape of thermal lesions^53^. The proximity, size, and blood flow rate play crucial roles in determining the extent of this effect^54–56^. This challenge is particularly pronounced in brain FUS applications, where precise thermal control is essential to avoid unintended damage to healthy surrounding tissues. We demonstrated in vivo lesioning capabilities well below the cortical surface (~1 cm), where OA imaging offered valuable structural information for circumventing vasculature within the focal zone. We were able to induce a highly localized thermal lesion, without disrupting functional vasculature or affecting surrounding tissue^57^.

While the exceptional resolution and label-free vascular sensitivity of our integrated OAgHIFU system enable precise targeting and monitoring of thermal lesion formation, its effective penetration depth remains confined to approximately 1–2 cm because of the strong light attenuation^58^. Despite the deeper reach of USgHIFU (>10 cm) or full-body MRgHIFU^10,59^, our system has potential for diverse therapeutic applications, particularly for the treatment of shallow liver tumors, subcutaneous lesions, and other localized pathologies where precise and controlled thermal ablation is required. Future studies should explore its adaptability to different anatomical regions, accounting for variations in tissue composition, vascularization, and treatment constraints.

Our system has the potential for inducing thermal lesions in a clinically relevant scenario, although further integration with functionalities like neuronavigation and image fusion with preoperative data needs to be implemented to meet current neurosurgery standards. Our approach enables brain vasculature imaging, FUS delivery, and a quantitative assessment of thermal lesion formation in the tissue around the focus that does not rely on temperature estimation. Furthermore, it provides real-time feedback on the lesion size by analyzing the OA signals acquired during sonication. This allows for continuous monitoring of lesion progression, enabling on-the-fly adjustments to the thermal dose and precise coordination of contiguous SPs to effectively sonicate larger volumes. Future directions of this work include integrating pulse-echo US imaging within the same device, together with Doppler ultrasound, thus providing complementary functional data by providing both structural visualization and hemodynamic parameters. Altogether, this technique opens a promising clinical avenue for new translational applications of HIFU treatments.

## Methods

### Optoacoustic guided HIFU system

OA imaging and HIFU sonication of the ex vivo mouse brain were performed using the OAgHIFU tomographic system (Fig. 1a). The system includes a custom-made matrix array probe (Imasonic SaS, France) featuring 512 individual elements. The array surface is hemispherical, with a 40 mm radius and 150° angular coverage (1.48 π solid angle). All elements, with a central frequency of 7 MHz and >80% bandwidth, were used for both volumetric OA data acquisition and HIFU emission. At the apex of the hemisphere, a central cavity (8 mm diameter), along with three lateral cavities (4 mm diameter, located at a 45° elevation angle and symmetrically spaced along the azimuthal angle), accommodate a custom-made optical fibers (CeramOptec GmbH, Bonn, Germany) for guiding the light beam. The OAgHIFU probe was adapted for in vivo sheep experiments (Fig. 5a) operating at a central frequency of 4 MHz with >45% bandwidth, and used for both volumetric OA data acquisition and HIFU therapy.

OA excitation was achieved using a pulsed optical parameter oscillator-based laser (SpitLight, Innolas Laser GmbH, Germany), delivering <10 ns pulses at 10 Hz repetition rate, with a tunable wavelength range of 680–1250 nm and pulse energies of approximately 180 mJ. Multi-directional illumination was implemented to ensure uniform light distribution, thereby improving image quality and detection sensitivity.

A custom-designed parallel high-speed digital acquisition (DAQ) system (Falkenstein Mikrosysteme, Germany) was employed for sampling the OA signals and controlling HIFU emission. The system was triggered via the Q-switch output of the laser, and data was transmitted to a dedicated PC via Ethernet for synchronization, system control and processing. OA signals were amplified by 40 dB and digitalized at 40 megasamples per second. The DAQ employed class D amplifiers (15 MHz bandwidth) capable of driving the array elements with digitally generated waveforms of arbitrary duration. The driving voltage was set to 6.5 Vp with transmission delays adjusted independently for each channel with a resolution of 5.5 ns, enabling precise beam steering to targeted focal locations. US beams were electronically steered by assigning appropriate transmission delays according to the time-of-flight to the intended focus.

OA images were acquired over a field of view (FOV) ranging from 10 × 10 × 10 mm^3^ to 30 × 30 × 20 mm^3^ (Supplementary Fig. 1) using a 128 × 128 × 128 voxel grid, corresponding to a spatial sampling of approximately 78 µm/voxel to 234 µm/voxel, respectively, and reconstructed using a GPU-accelerated back-projection algorithm^60^. Prior to reconstruction, the signals were band-pass filtered between 0.1 MHz and 3 MHz. All data processing was conducted in MATLAB (R2023a, MathWorks, USA) using custom-developed software unless otherwise stated.

### System characterization

We measured the array’s pressure using a calibrated hydrophone (Precision Acoustics, UK) in a degassed water tank. To avoid damaging the hydrophone, we measured its focus at low pressure and then measured the pressure out of focus, increasing the voltage to extrapolate the peak pressure. Under these operating conditions, the array achieves a peak pressure of 6.8 MPa when driven at ±6.5 V and 5 MHz, corresponding to a spatial peak time average intensity (ISPTA) of 716 W/cm^2^ at a 49% duty cycle and a mechanical index MI = 3. The peak heating power density, assuming an attenuation coefficient of *ɑ* = 0.92 cm^−1^ (*ɑ₀* = 0.8 dB/(cm MHz) for brain tissue)^61^, is calculated to be 1.3 kW/cm^3^.

The US focus at 5 MHz is ellipsoidal (Fig. 2a). The spatial extent of the pressure field, quantified by its full width at half maximum (FWHM), is 464 µm axially and 205 µm laterally. We calculated the ellipticity of the focus, *f*, as follows:

1 $$f=({{FWHM}}_{{axial}}-{{FWHM}}_{{lateral}})\,/\,{{FWHM}}_{{axial}}$$

which results in a value of *f* = 0.56. To characterize the system’s OA imaging spatial resolution, the PSF was measured by imaging a single ≈30 µm microsphere (Fig. 2a). This yielded axial and lateral resolutions of 101 and 117 µm, respectively, as defined by the FWHM.

### Animal model

All experiments were performed in accordance with the Swiss Animal Welfare Act and were approved by the Cantonal Veterinary Office Zurich. A total of 10 female wild-type C57BL/6J mice (*Mus musculus*), aged 8–12 weeks, were used in this study. The mice were obtained from The Jackson Laboratory, strain #000664, and were not genetically modified. All mice were housed in ventilated cages inside a temperature-controlled room under a 12-h dark/light cycle. The temperature was 21 ± 1 °C, with a relative humidity of 55 ± 10%. Pelleted food and water were provided ad libitum. A single adult female White Alpine Sheep (Ovis aries), aged 3 years and 7 months, was included in this study (animal license number ZH109/2024). The animal originated from a Maedi-Visna-free herd, as confirmed by prior serological testing. The source herd was routinely vaccinated against Pasteurella and Clostridia and followed a regular deworming protocol under veterinary supervision. Ongoing health management included a general physical examination every 4 weeks and hoof trimming every 8 weeks.

The sheep was transported in accordance with animal welfare regulations. Following arrival, it underwent a minimum acclimatization period of 7 days. A veterinarian then performed a baseline physical examination and recorded the body weight. Only animals confirmed to be healthy, non-pregnant, and displaying normal clinical parameters were enrolled in the study.

Prior to surgery, pregnancy was ruled out using a rapid visual pregnancy test (Idexx Alertys) combined with an ultrasound examination. As part of the pre-anesthetic assessment, a 10–15 mL blood sample was collected from the vena jugularis via a Vacutainer system for a complete blood count and serum chemistry panel.

### Anesthesia protocol

All animals were anesthetized according to standardized protocol. Mice were euthanized under deep anesthesia (5% isoflurane for 5 min).

The sheep received a standardized premedication regimen after a 24-h fast, prior to anesthesia induction. Intramuscular buprenorphine (0.01 mg/kg BW im, 0.03 mL/kg) and medetomidine (0.005–0.01 mg/kg BW) were administered 30–45 min beforehand. Following intravenous catheter placement, carprofen (4 mg/kg BW, 0.08 mL/kg BW) was given for pre-emptive analgesia. Induction was achieved intravenously using midazolam (0.1 mg/kg BW, 0.02 mL/kg BW), ketamine (3–5 mg/kg BW, 0.03–0.05 mL/kg BW), and propofol (0.4–4 mg/kg BW, 0.04–0.4 mL/kg BW), titrated to effect. Maintenance of anesthesia employed a balanced protocol of inhaled isoflurane (1–3%) with oxygen and air (FiO_2_ 50–70%), supplemented by intravenous infusions of propofol (0.5–1 mg/kg/h, 0.05–0.1 mL/kg/h) and ketamine (1.2–3 mg/kg/h, 0.012–0.03 mL/kg/h).

After tracheal intubation confirmed by capnography, supportive measures were implemented. These included the application of ophthalmic ointment and continuous intravenous fluid therapy with Ringer’s lactate at 5–10 mL/kg/h. Comprehensive physiological monitoring was performed throughout, encompassing electrocardiogram, heart rate, invasive arterial blood pressure, capnography, pulse oximetry, and esophageal temperature. All monitored parameters were recorded at 10-min intervals.

### Rodent preparation and integration in the setup

The skin covering the head was carefully removed to expose the underlying skull structure, ensuring minimal disruption to surrounding tissues. Following this, the skull was meticulously removed to allow direct access to the brain. This technique was performed with precision to maintain the integrity of the targeted area and facilitate accurate intervention. Then, the mouse was placed in a manual multi-axis stage (Thorlabs, United States), thus allowing fine three-dimensional positioning of the mouse head for subsequent OA imaging and navigation of the brain vasculature. Finally, the head of the mouse was fixed into a custom-designed stereotactic mouse head holder (Narishige International, Japan).

The curved array was positioned with the concave volume facing downwards, and put within a 3D printed structure filled with degassed water. The curved concave volume was thus filled to ensure proper ultrasound signal coupling. An aperture at the bottom of the bath served as a transmission window and was isolated from the exterior with transparent plastic film. For definitive positioning of the mouse, we placed the head below the aperture, centered to the curved array. A large portion of degassed ultrasound gel fills the gap between the brain and the film window, which was previously put on top of the mouse brain, thus leaving no air in between. We gently moved the mouse brain toward the foil window, taking care not to press it against the surface. For perfect alignment of the mouse head with respect to the OA imaging FOV, we run a real-time pre-visualization and adjust the position of the mouse head in all axes using the multi-axis stage.

### Large animal preparation and integration in the setup

The initial MRI scan, conducted to provide detailed brain morphology, was performed 30 min after anesthetic induction (“Methods”). This was complemented by a CT scan to evaluate cranial thickness and geometry, ensuring procedural safety. Following imaging, the sheep was positioned prone on the surgical table with its limbs secured and its head immobilized within a custom stereotactic frame. A bilateral craniotomy over the frontal and parietal lobes was performed to provide transducer access, exposing the underlying dura mater. All subsequent experimental steps were carried out prior to dural incision; the dura was then opened bilaterally in a C-shaped pattern using microscissors.

For combined OA and HIFU procedures, the brain surface was prepared to ensure optimal coupling. A mixture of ultrasound gel and phosphate-buffered saline (PBS) was centrifuged to remove air bubbles and applied to the exposed cortex. A 3D-printed frame surrounded the brain, holding a transparent, degassed water-filled bag, contacting the brain surface. A temperature-controlled silicone coil around the frame maintained the medium at 37 °C to prevent brain cooling. Fine probe position adjustments were made using a multi-axis stage, and real-time OA imaging was used for target visualization and depth selection prior to treatment.

To evaluate the performance of the OAgHIFU device, a post-treatment MRI was performed to assess the induced lesions using an intravenous gadolinium-based contrast agent (DOTAREM, 0.2 mL/kg (0.1 mmol/kg)). Following the procedure, the animal was euthanized (pentobarbital, 150 mg/kg bodyweight, 0.5 mL/kg, i.v.). Death was confirmed by the absence of heartbeat and pupillary reflex. Brain tissue was subsequently harvested for histological analysis to determine the extent of the targeted lesions and to identify any concomitant adverse effects, particularly micro- or macroscopic hemorrhage in off-target regions.

### MRI scanning and image processing

MRI was used to validate the coagulated regions in the brains of mice and sheep. In the mouse model, all MRI images for post-FUS validation were acquired by a horizontal bore small animal 7 T MRI scanner (PharmaScan, Bruker BioSpin, Germany) using a CryoProbe with the software ParaVision 6.0.1. Adjustments to the basic frequency, reference power and shimming were performed before the data acquisition. We used a high-resolution 2D RARE sequence to acquire 16 sagittal slices, with the following parameters: repetition time of 4000 ms, echo time of 11.2337 ms, FOV of 16 × 16 mm^2^, matrix size of 267 × 267, resolution of 60 × 60 μm^2^, slice thickness of 0.4 mm, slice gap of 0 mm, RARE factor of 6, average of 8. Subsequently, we used a 2D RARE sequence to acquire 20 coronal slices with high resolution using the following parameters: repetition time of 4000 ms, echo time of 59.84 ms, echo spacing of 19.946 ms, bandwidth of 32894.7 Hz, FOV of 10 × 12.7 mm^2^, matrix size of 400 × 512, resolution of 25 × 25 μm^2^, slice thickness of 0.4 mm, slice gap of 0.1 mm, RARE factor of 8, and average of 16. MRI data analysis and visualization were done using the AFNI software (Analysis of Functional NeuroImages, NIH, USA). Command “to3d” was used to convert imaging data from the BRUKER format to NIfTI format, then the “afni&” command was used to visualize and save the images to Tagged Image File Format (TIFF). The relevant source codes for AFNI can be downloaded freely through https://afni.nimh.nih.gov/afni/.

For the sheep study, MRI data were acquired on a 3 Tesla clinical scanner (Philips Elition 3.0 T X, Philips AG, Zurich, Switzerland) equipped with a 32-channel head coil (dStream HeadSpine solution, Philips AG). Animals were positioned head-first in a supine orientation. For baseline anatomical evaluation and neuronavigation, two volumetric sequences were performed: a 3D T1-weighted TFE sequence (TR of 10 ms, TE of 4.6 ms, flip angle of 8°, FOV of 210 × 210 × 176 mm^3^, isotropic voxel size of 0.8 mm, acquisition time of 06:07 min) and a 3D T2-weighted TSE sequence (TR of 2500 ms, TE of 300 ms, flip angle of 90°, FOV of 250 × 250 × 174 mm^3^, isotropic voxel size of 0.8 mm, acquisition time of 06:40 min). Following the therapeutic intervention, the identical pre-treatment protocol was repeated and expanded to include several supplementary sequences optimized for post-treatment lesion detection. These additional sequences consisted of proton density-weighted turbo spin echo (PDW-TSE) scans in coronal (TR of 2822 ms, TE of 30 ms, refocusing flip angle of 90°, FOV of 160 × 120 × 171 mm^3^, voxel size of 0.50 × 0.50 × 3.00 mm^3^, acquisition time of 03:40 min) and transversal (TR of 2500 ms, TE of 30 ms, refocusing flip angle of 90°, FOV of 170 × 170 × 135 mm^3^, voxel size of 0.40 × 0.43 × 3.00 mm^3^; acquisition time of 03:15 min) orientations, a sagittal PDW-TSE sequence with spectral presaturation with inversion recovery (SPAIR) for fat suppression (TR of 3567 ms, TE of 30 ms, refocusing flip angle of 90°, FOV of 170 × 175 × 152 mm^3^, voxel size 0.42 × 0.53 × 3.00 mm^3^, acquisition time of 06:36 min), and a transversal fluid attenuated inversion recovery (FLAIR) sequence (TR of 11,000 ms, TE of 125 ms, TI of 2800 ms, refocusing angle of 120°, FOV of 200 × 200 × 115 mm^3^, voxel size of 0.45 × 0.62 × 3.00 mm^3^, acquisition time of 04:46 min). MRI data analysis and visualization were done using 3D Slicer 5.10^62^.

### Histology

The mice brains were extracted after MRI scans and were fixed overnight in 4% paraformaldehyde to assess FUS-induced thermal lesions. Then, the brains were equilibrated in 15 and 30% sucrose solutions prepared in 0.1 M PBS at 4 °C. Brains were sectioned into 2–3 μm-thick slices using a microtome (HM355 S, Thermo Scientific, United States). Free-floating sections were washed with PBS, mounted onto microscope slides, and stained with H&E in a Sakura Finetek Tissue-Tek Prisma stainer. Wide-field images were captured using a digital slide scanner NanoZoomer-XR C12000 (Hamamatsu, Hamamatsu City, Japan) to evaluate H&E staining. Digital images were minimally processed in Lightroom Classic 15.3.1 (Adobe) to adjust brightness, contrast and subtract background for visualization purposes.

In the sheep model, the brain was carefully extracted from the cranial cavity post-euthanasia for subsequent processing. Tissue was fixed via immersion in 10% neutral buffered formalin for subsequent histological analysis. After fixation, specimens were processed according to standard histological protocols to preserve tissue architecture and cellular morphology. Coronal sections of 2–3 µm thickness were cut using a microtome (HM355 S, Thermo Scientific, USA). Free‑floating sections were mounted onto glass slides and stained with H&E using a Sakura Finetek Tissue‑Tek Prisma automated stainer. Whole‑slide imaging was performed with a NanoZoomer 2.0‑HT digital slide scanner (Hamamatsu, Japan). Brightness, contrast, and background were minimally adjusted in Adobe Lightroom Classic 15.3.1 for optimal visualization.

### OA signal analysis

For comparison purposes, we normalized the OA data from the different experiments to a common reference. Thus, for each SP, we first normalized the temporal OA signal of each voxel relative to the voxel at the focus. We then subtracted the median baseline value, ensuring that the relative change starts from 0 at the onset of HIFU. After computing the relative change in OA with respect to the baseline (ΔOA/OA) for every voxel, we discarded those voxels that exhibited a decrease in this value during the first few seconds into sonication. Since such decay is not physically plausible, we attributed it to artifacts arising from the partial angular coverage of the transducer.

For the analysis of the OA signals, we defined various VOIs. These VOIs are structured as ellipsoidal shells centered around the focal point. To define each VOI, we specified inner and outer distances within the horizontal plane of the brain (xy plane, Fig. 3a). Next, we calculated inner and outer distances along the z-axis by scaling the horizontal distances by a factor equal to the ratio of the HIFU focus dimensions (FWHM_axial_/FWHM_lateral_, Figs. 2a and 3b) defined as *h* and equal to ~2.26. This approach enabled the creation of concentric ellipsoidal shells that resemble the geometry of the HIFU focus. We used these VOIs to calculate the mean ΔOA/OA trace by averaging the ΔOA/OA values of the voxels contained by them. The dimensions of *R*_1_ and *R*_2_ (Fig. 3b) used for the OA signal analysis of the in vivo data (Fig. 5b) are: 0–0.5 mm for VOI_1_, 0.5–1 mm for VOI_2_, 1–1.5 mm for VOI_3_, and 1.5–2 mm for VOI_4_.

### OA and MRI image registration

MRI and OA mice brain data were registered using a manual, feature-based method. Initially, the OA dataset underwent geometric transformations and rotations around major axes to align with the corresponding MRI images. The midline of the brain served as the primary reference point for establishing correspondence between the two imaging modalities. The superior sagittal sinus was systematically registered across both datasets for spatial orientation along the transverse plane. In the coronal plane, alignment was facilitated by identifying and matching the skull boundaries visible in both data sets. OA and MRI datasets were rescaled to account for differences in pixel-to-millimeter resolution ratios, enabling overlay and integration of the imaging data.

### Quantitative analysis of lesion dimension

For estimating the range of the lesion size using OA data (*V*_*L*, *range*_, Fig. 4e), we have first assessed which is the outermost VOI (OM-VOI, Fig. 4e right), which OA analysis reveals as lesioned. For this, we considered all the VOIs composing a single SP. Thus, we took the average ΔOA/OA trace of every VOI and fit them to an exponential curve. Then, we calculated the coefficient of determination *R*^2^, which measures the proportion of the variance in the dependent variable that is predictable from the independent variable. We have done this to identify which VOIs are subjected to thermal lesion (OA signal exponential increase interrupted by changes in optical properties, Fig. 3c top row) from VOIs subject to mere heating (OA signal exponential increase during sonication period, Fig. 3c bottom row). Given that this value spans from 0 to 1 (the closer to 1, the better the exponential fitting), we defined a threshold of 0.95. Thus, VOIs with smaller values of *R*^2^ are indicative of thermal lesion, and VOIs with larger values of *R*^2^ are not. Following this, we determined the OM-VOI indicative of the thermal lesion extension. Since a VOI is represented as an ellipsoidal shell (Fig. 3b), we estimated the lesion size within the volume range that spans between the volume covered by the inner (*V*_*in*_) and the outer (*V*_*out*_) layers of the OM-VOI (Fig. 4e, right). For this approximation, we have assumed that the morphology of the lesion will approximate the morphology of the HIFU focus (Fig. 2a), and therefore assumed 3D ellipsoidal volumes.

For calculating the lesion size (*V*_*L*_), we analyzed the H&E histological sections of 7 lesions. Among these are included the lesions caused in the SPs (Fig. 3a) plus four lesions from two additional experiments performed in different mice. We applied the following procedure to each lesion: for all the histological sections obtained from a given lesion, we began with quantitative contrast enhancement, followed by an intensity-based segmentation approach. To enhance the contrast between the lesion and healthy tissue, we leveraged the observation that cells inside and outside the lesion differ in water content around the nuclei (Fig. 4c). Water appears in the histological sections as white, corresponding to high pixel intensity. We established an intensity threshold (*I*_*th*_) that separates water from the rest of the intensity levels representing the H&E staining. Then, for each pixel, we calculated the number of neighboring pixels with intensities greater than *I*_*th*_ using a 5 × 5 interrogating window. This formed the basis for the subsequent segmentation of the lesion (Fig. 4d). Next, we applied a threshold to convert the image into a binary image. The resulting image was cleaned by applying sequential erosion and dilation steps, followed by filling small holes. Once the lesion region achieved homogeneity, we extracted its contour. Ultimately, we used Fourier contour analysis to obtain a smooth approximation of the contour^63^. We used principal component analysis (PCA) to extract the principal axis of the approximated contour, from which we derived its height and width (Fig. 4f). Next, we selected the histological section represented by the largest lesion height and width. Assuming symmetry of the lesion along its principal axis (PA_1_, Fig. 4f), we estimated its volume by calculating the volume of revolution.

To summarize the discrepancy between *V*_*L*, *range*_ and *V*_*L*_, we used the midpoint of each *V*_*L*, *range*_ range as a simple scalar summary statistic. For each SP, this midpoint was calculated as the average of the lower and upper bounds of the corresponding *V*_*L*, *range*_, and the discrepancy was defined as the midpoint-based *V*_*L*, *range*_ estimate minus *V*_*L*_. We then calculated the mean discrepancy and the mean absolute discrepancy across all SPs. Because the OA framework provides a bounded range rather than a single point estimate, this midpoint-based discrepancy was used only as an approximate descriptive metric.

For the in vivo sheep experiment, the OA signal analysis shown in Fig. 5b was performed using concentric ellipsoidal VOIs spaced by 250 µm in the radial direction. For each VOI, we extracted the minimum derivative value during sonication and used a negative derivative threshold to identify the abrupt OA downturn associated with coagulation. The outermost VOI satisfying this criterion was then used to define the refined lesion-width estimate. In the present study, the derivative threshold was set to −5 %/s, resulting in a lesion width of 1–1.5 mm wide.

### Reporting summary

Further information on research design is available in the Nature Portfolio Reporting Summary linked to this article.

## Supplementary information

Supplementary Information

Description of Additional Supplementary Files

Supplementary Movie 1

Reporting Summary

Transparent Peer Review File

## Source data

Source Data

## Data Availability

Source data generated in this study are provided with this paper. Additional raw imaging data are too large to be publicly shared. Access can be requested from the corresponding authors. Requests will be reviewed by the authors and the relevant institution, and access may be granted for non-commercial research purposes subject to institutional approval and an appropriate data-use agreement. A response is expected within 30 days. Once granted, access will be provided for 12 months, with extensions subject to renewed approval. Source data are provided with this paper.

## Code availability

The custom code is not publicly deposited because it contains sensitive technical information and is subject to institutional restrictions on public dissemination. Access can be requested from the corresponding authors. Requests will be reviewed by the authors and the relevant institution, and access may be granted for non-commercial research purposes, subject to institutional approval and an appropriate software-use agreement. A response is expected within 30 days. Once granted, access will be provided for 12 months, with extensions subject to renewed approval.
