# Supplementary material for: Integrated device for optoacoustically-guided ultrasound surgery
Source: Nat Commun. 2026 Jul 21;17:8871. doi: 10.1038/s41467-026-75692-4 (PMC13500822; doi:10.1038/s41467-026-75692-4)
Supplement: Supplementary file 1 [file 41467_2026_75692_MOESM1_ESM.pdf]

Supplementary Information

Integrated device for optoacoustically-guided ultrasound surgery

Isaac Esteban[1,2], Nima Mahkam[1,2], Yi Chen[1,2], Martha Gjikolaj[2,3], Beat Werner[4], Henning Richter[5], Katrin Beckmann[5], Edin Nevzati[6,7], Daniel Coluccia[8], Hikari A. I. Yoshihara[1,2], Frauke Seehusen[9], Daniel Razansky[1,2]*, and Hector Estrada[1,2]*

[1]Institute for Biomedical Engineering, University of Zurich and ETH Zurich, 8093, Switzerland

[2]Institute of Pharmacology and Toxicology, University of Zurich, 8057, Switzerland

[3]Brain Research Institute, University of Zurich, 8057, Switzerland

[4]Center for MR-Research, University Children's Hospital Zurich, 8032, Switzerland

[5]Clinic for Diagnostic Imaging, Diagnostic Imaging Research Unit (DIRU), University of Zurich, Zürich, Switzerland

[6]University of Basel, Faculty of Medicine, Basel, Switzerland, Klingelbergstrasse 61, 4056 Basel

[7]Department of Neurosurgery, University of Colorado Anschutz Medical Campus School of Medicine, 1635 Aurora, Aurora, CO 80045, USA

[8]Neurosurgery, Stadtspital Zurich, Birmensdorferstrasse 497, 8063, Zurich, Switzerland

[9]Laboratory for Animal Model Pathology (LAMP), Institute of Veterinary Pathology, Vetsuisse Faculty, University of Zurich, 8057, Switzerland

Supplementary Figures

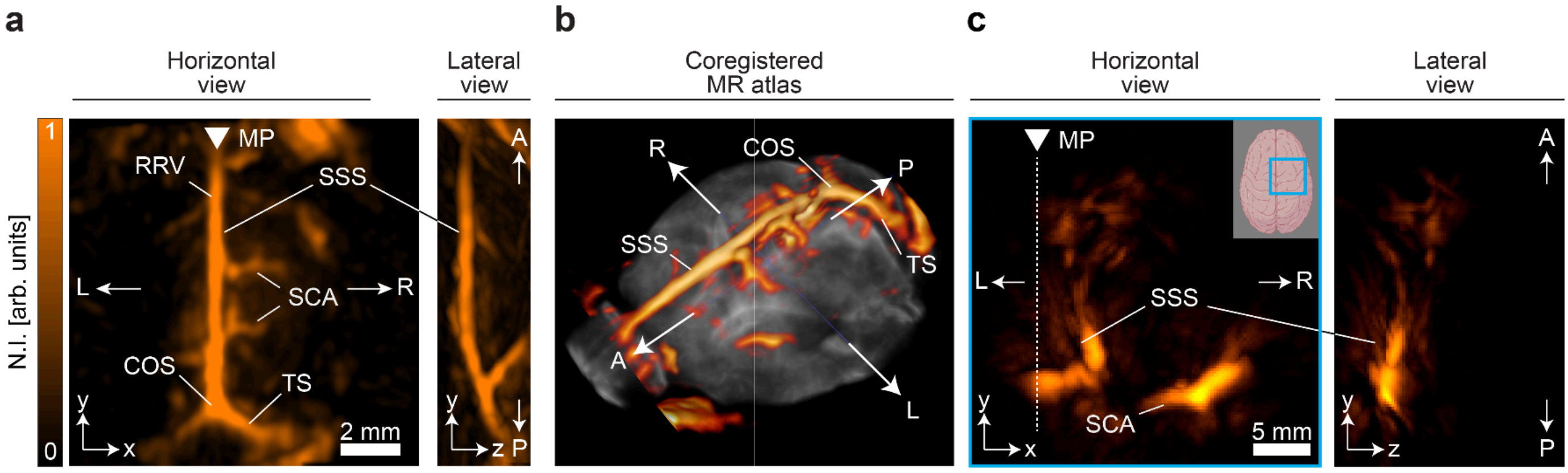

Supplementary Figure 1. **OA imaging at 780 nm reveals blood vessels and vascular structures in the mouse and sheep brain**. **a**. OA image reconstruction representing a single time point from the pre-sonication phase (Fig. 1b) of the experiments, acquired using a laser source wavelength of 780 nm. The different windows show the maximum intensity projections (MIPs) aligned with the horizontal and sagittal views. The white arrow indicates the position and direction of the midplane (MP). The imaged FOV is 12 × 12 × 12 mm^3. **b**. 3D reconstruction of the vascular network from **a**, registered to an MR atlas. **c**. Volumetric OA MIPs of the sheep brain (FOV is 30 × 30 × 20 mm^3). The inset shows a top-view brain schematic indicating the approximate location of the OA imaging FOV. The top-view brain schematic in the inset was created in BioRender. Mahkam, N. (2026) https://BioRender.com/tciyd5c and adapted by the authors using Adobe Illustrator 2026. The white arrow indicates the position and direction of the MP. Superior sagittal sinus (SSS); Transverse sinus (TS); Superior cerebellar arteries (SCA); Confluence of sinuses (COS); Rostral rhinal veins (RRV); Left (L); Right (R); Anterior (A); Posterior (P).

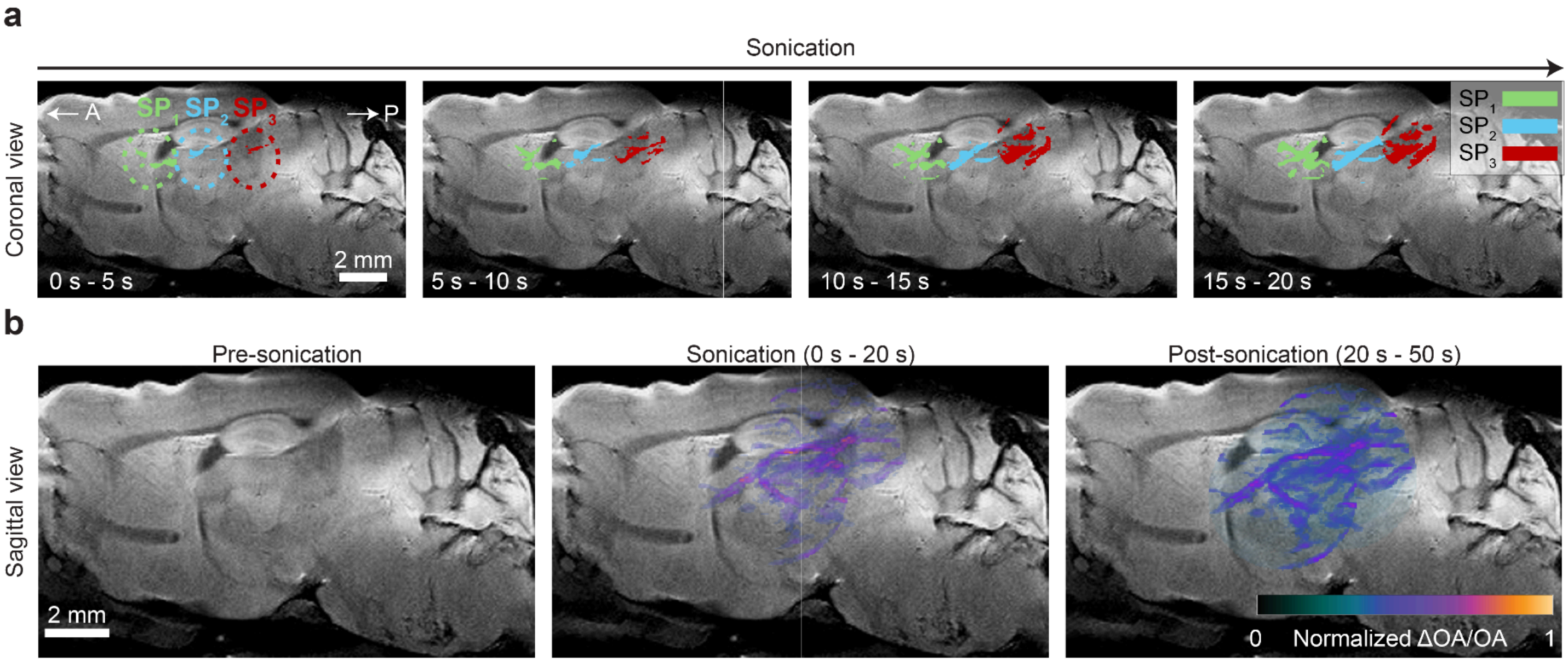

Supplementary Figure 2. **MRI-registered visualization of OA changes during FUS sonication**. **a**. Relative optoacoustic signal change (ΔOA/OA) overlaid on T2-weighted MRI sections after OA–MRI co-registration. Representative time points during sonication illustrate the temporal evolution of the OA response around SP1, SP2 and SP3, each represented with distinct colors. For visualization, OA data were temporally high-pass filtered (6 %) to emphasize dynamic signal changes. **b**. Normalized maps of relative OA change for all three sonication experiments (SP_1, SP_2, and SP_3) during different phases: pre-sonication, sonication (0 s - 20 s), and post-sonication (20 s - 50 s). Each heat map shows the maximum OA intensity change observed within the corresponding time window. OA data are spatially and temporally normalized and processed with a 6 % temporal high-pass filter.

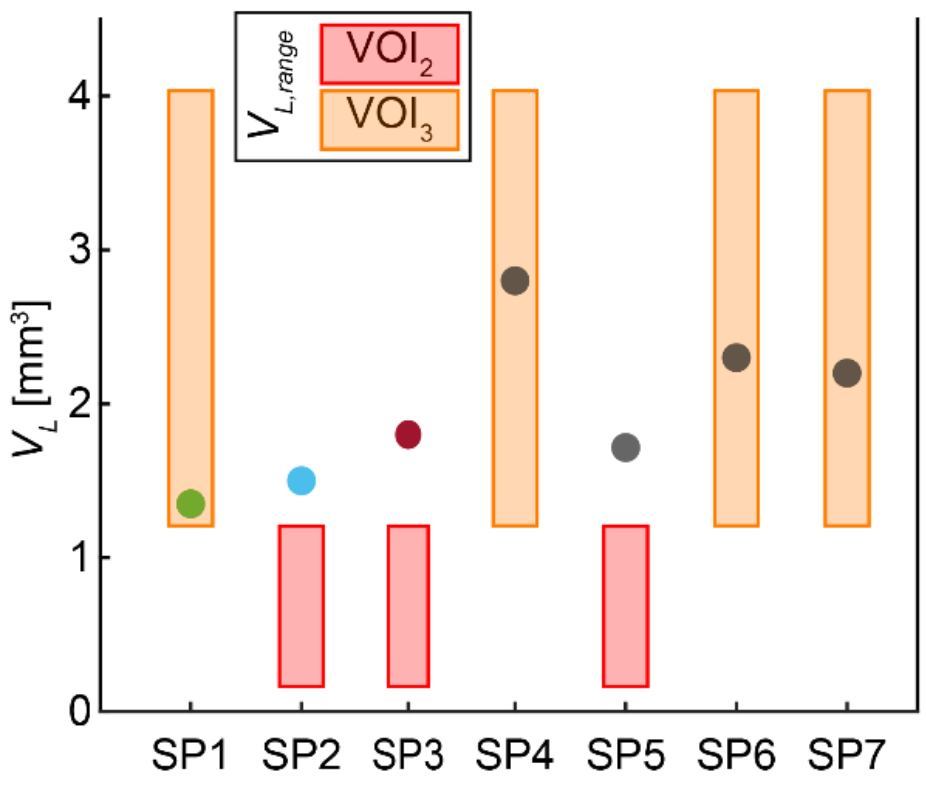

Supplementary Figure 3. Lesion-by-lesion comparison between OA-based lesion-volume range estimation and histology-derived lesion volume. For each sonication point (SP), the vertical range bar indicates the lesion-volume range estimated from OA analysis ($V_{L,range}$, Fig. 4e). Red bars correspond to lesions assigned to VOI_2 and orange bars correspond to lesions assigned to VOI_3. Dots indicate the corresponding histology-derived lesion volume (V_L, Fig. 4d) quantified from H&E-stained sections. Colored dots correspond to SP_1, SP_2 and SP_3 shown in Fig. 3a, and gray dots correspond to additional SPs. Data are shown for n = 7 independent thermal lesions.

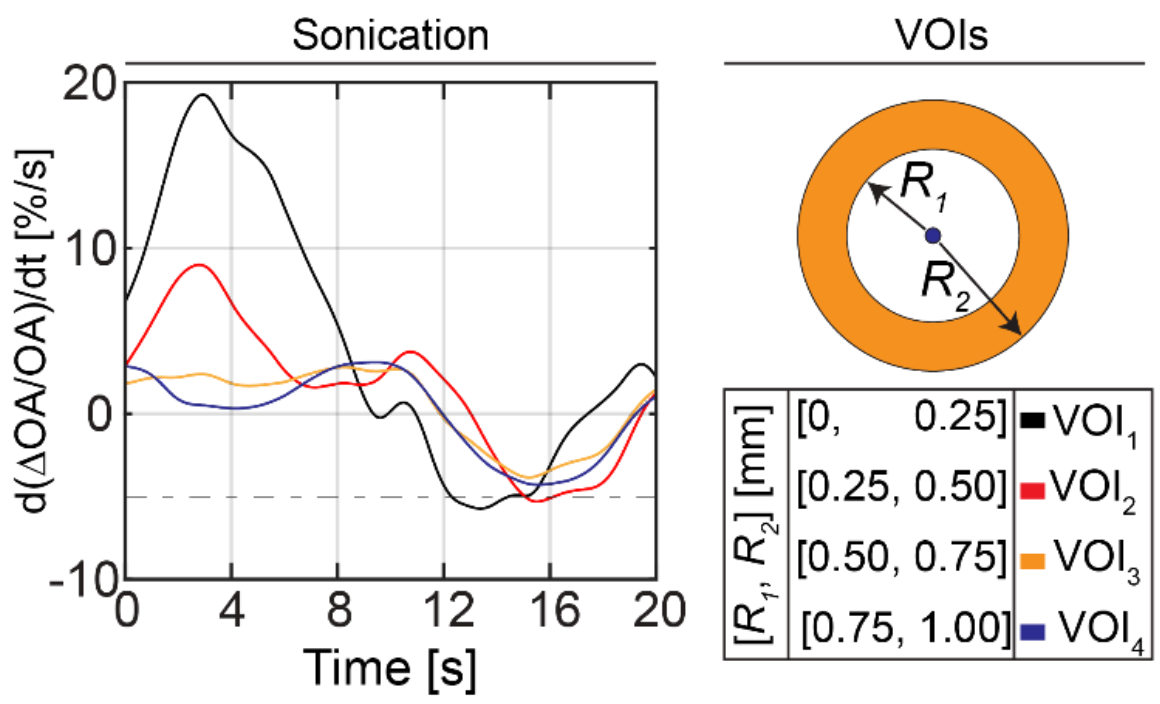

Supplementary Figure 4. Temporal derivatives of ΔOA/OA during sonication in the in vivo sheep experiment. Temporal derivatives of the relative OA signal change (ΔOA/OA) for the concentric ellipsoidal VOIs analyzed in the in vivo sheep experiment, shown over the sonication time window only. The curves were used to refine lesion-size estimation within the lesion-associated region identified in Fig. 5b. The horizontal dashed line indicates the derivative threshold used to identify the abrupt signal downturn associated with coagulation of -5 %/s. Data are from the single in vivo sheep experiment (n = 1 animal). Colour-coded curves correspond to VOI_1–VOI_4 as indicated in the inset.

Supplementary Movie 1. **OA visualization of a FUS-induced lesion**. Visualization of the OA activation regions corresponding to SP_2 (Fig. 3a). Scale bar 1 mm.
