# Supplementary material for: Integrated device for optoacoustically-guided ultrasound surgery
Source: Nat Commun. 2026 Jul 21;17:8871. doi: 10.1038/s41467-026-75692-4 (PMC13500822; doi:10.1038/s41467-026-75692-4)
Supplement: Supplementary file 2 [file 41467_2026_75692_MOESM2_ESM.pdf]

Description of Additional Supplementary Files

Supplementary Movie 1.

Optoacoustic visualization of focused ultrasound-induced lesion. Relative optoacoustic signal change (ΔOA/OA, colorscale) overlaid on T2-weighted MRI sections (gray) after OA–MRI co-registration. Scale bar: 1 mm.
