# Supplementary material for: Integrated device for optoacoustically-guided ultrasound surgery
Source: Nat Commun. 2026 Jul 21;17:8871. doi: 10.1038/s41467-026-75692-4 (PMC13500822; doi:10.1038/s41467-026-75692-4)
Supplement: Supplementary file 3 [file 41467_2026_75692_MOESM4_ESM.pdf]

Corresponding author(s): Hèctor Estrada

Last updated by author(s): May 12, 2026

Reporting Summary

Nature Portfolio wishes to improve the reproducibility of the work that we publish. This form provides structure for consistency and transparency in reporting. For further information on Nature Portfolio policies, see our Editorial Policies and the Editorial Policy Checklist.

Statistics

For all statistical analyses, confirm that the following items are present in the figure legend, table legend, main text, or Methods section.

n/a	Confirmed	
☐	☒	The exact sample size (n) for each experimental group/condition, given as a discrete number and unit of measurement
☐	☒	A statement on whether measurements were taken from distinct samples or whether the same sample was measured repeatedly
☒	☐	The statistical test(s) used AND whether they are one- or two-sided<br>*Only common tests should be described solely by name; describe more complex techniques in the Methods section.*
☒	☐	A description of all covariates tested
☒	☐	A description of any assumptions or corrections, such as tests of normality and adjustment for multiple comparisons
☐	☒	A full description of the statistical parameters including central tendency (e.g. means) or other basic estimates (e.g. regression coefficient) AND variation (e.g. standard deviation) or associated estimates of uncertainty (e.g. confidence intervals)
☒	☐	For null hypothesis testing, the test statistic (e.g. F, t, r) with confidence intervals, effect sizes, degrees of freedom and P value noted<br>*Give P values as exact values whenever suitable.*
☒	☐	For Bayesian analysis, information on the choice of priors and Markov chain Monte Carlo settings
☒	☐	For hierarchical and complex designs, identification of the appropriate level for tests and full reporting of outcomes
☒	☐	Estimates of effect sizes (e.g. Cohen's d, Pearson's r), indicating how they were calculated

Our web collection on statistics for biologists contains articles on many of the points above.

Software and code

Policy information about availability of computer code

Data collection

Focused ultrasound emission and optoacoustic signal acquisition were performed using a dedicated data acquisition (DAQ) system controlled through a custom MATLAB interface (MATLAB R2023a, MathWorks, Natick, MA, USA). MATLAB was used to configure the DAQ hardware and manage data flow during experiments. Mouse MRI data were acquired using ParaVision 6.0.1 (Bruker BioSpin). Sheep MRI data were acquired using the Philips MRI system software. Histological whole-slide images were acquired using the software associated with the NanoZoomer-XR C12000 and NanoZoomer 2.0-HT scanners.

Data analysis

Optoacoustic data acquisition and HIFU emission were controlled through a custom MATLAB interface (MATLAB R2023a, MathWorks). Signal processing and image reconstruction were also performed in MATLAB R2023a using custom scripts implementing the methods described in the manuscript. These steps included standard preprocessing and reconstruction procedures, band-pass filtering, GPU-accelerated back-projection reconstruction, extraction of signal intensity changes from defined volumes of interest, curve fitting, derivative-based analysis and lesion-size estimation.
Mouse MRI data were acquired using ParaVision 6.0.1 (Bruker BioSpin) and processed/visualized using AFNI, including conversion from Bruker format to NIfTI using to3d. Sheep MRI data were acquired using the Philips MRI system software and analysed/visualized using 3D Slicer 5.10. Histological whole-slide images were acquired using the software associated with the NanoZoomer-XR C12000 and NanoZoomer 2.0-HT scanners. Brightness, contrast and background adjustments of histological images were performed using Adobe Lightroom Classic 15.3.1.
Statistical analysis was limited to descriptive measures and quantitative summaries, including mean values, standard deviation, range-based lesion-size estimates and lesion-by-lesion discrepancies. No hypothesis-driven statistical comparisons or inferential statistical tests were performed.

For manuscripts utilizing custom algorithms or software that are central to the research but not yet described in published literature, software must be made available to editors and reviewers. We strongly encourage code deposition in a community repository (e.g. GitHub). See the Nature Portfolio guidelines for submitting code & software for further information.

Data

Policy information about availability of data

All manuscripts must include a data availability statement. This statement should provide the following information, where applicable:

- Accession codes, unique identifiers, or web links for publicly available datasets
- A description of any restrictions on data availability
- For clinical datasets or third party data, please ensure that the statement adheres to our policy

Source Data generated in this study are provided with this paper. Additional raw imaging data are too large to be publicly shared. Access can be requested from the corresponding authors. Requests will be reviewed by the authors and the relevant institution, and access may be granted for non-commercial research purposes subject to institutional approval and an appropriate data-use agreement. A response is expected within 30 days. Once granted, access will be provided for 12 months, with extensions subject to renewed approval.

Research involving human participants, their data, or biological material

Policy information about studies with human participants or human data. See also policy information about sex, gender (identity/presentation), and sexual orientation and race, ethnicity and racism.

Reporting on sex and gender	This study did not involve human participants, human data, or human biological materials.
Reporting on race, ethnicity, or other socially relevant groupings	This study did not involve human participants, human data, or human biological materials.
Population characteristics	This study did not involve human participants, human data, or human biological materials.
Recruitment	This study did not involve human participants, human data, or human biological materials.
Ethics oversight	This study did not involve human participants, human data, or human biological materials.

Note that full information on the approval of the study protocol must also be provided in the manuscript.

Field-specific reporting

Please select the one below that is the best fit for your research. If you are not sure, read the appropriate sections before making your selection.

☒ Life sciences ☐ Behavioural & social sciences ☐ Ecological, evolutionary & environmental sciences

For a reference copy of the document with all sections, see nature.com/documents/nr-reporting-summary-flat.pdf

Life sciences study design

All studies must disclose on these points even when the disclosure is negative.

Sample size	No formal statistical sample size calculation was performed. The number of animals was determined empirically based on the requirements for technical validation and feasibility assessment of the integrated focused ultrasound and optoacoustic imaging system. Experiments were conducted until consistent lesion induction and imaging performance were demonstrated across animals. The study was not designed to test a specific biological hypothesis or to perform inferential statistical comparisons between groups.
Data exclusions	No data was excluded from the analysis.
Replication	Key findings were replicated across independent animals. Thermal lesion formation and the associated optoacoustic signal changes were observed consistently in all animals included in the study. These repetitions represent biological replicates demonstrating reproducibility of the system’s performance. The study focused on technical feasibility rather than formal hypothesis testing; therefore, no predefined number of repeated trials per condition was established.
Randomization	No randomization procedures were used in this study. Animals were not allocated to different experimental groups, as the objective was to evaluate the technical performance and feasibility of the integrated focused ultrasound and optoacoustic imaging system. Each experiment consisted of targeted lesion induction followed by imaging and histological verification. Therefore, random group assignment was not relevant to the study design.
Blinding	No blinding procedures were implemented in this study. The same operators were responsible for focused ultrasound sonication, optoacoustic imaging, and subsequent data analysis. Blinding was not considered essential because the study objective was to evaluate the technical feasibility and performance of the integrated system rather than to compare outcomes between experimental groups. Lesion formation was assessed using objective imaging metrics and confirmed by magnetic resonance imaging and histological analysis, which minimized the risk of subjective bias in outcome interpretation.

Reporting for specific materials, systems and methods

We require information from authors about some types of materials, experimental systems and methods used in many studies. Here, indicate whether each material, system or method listed is relevant to your study. If you are not sure if a list item applies to your research, read the appropriate section before selecting a response.

Materials & experimental systems

n/a	Involved in the study
☒	☐ Antibodies
☒	☐ Eukaryotic cell lines
☒	☐ Palaeontology and archaeology
☐	☒ Animals and other organisms
☒	☐ Clinical data
☒	☐ Dual use research of concern
☒	☐ Plants

Methods

n/a	Involved in the study
☒	☐ ChIP-seq
☒	☐ Flow cytometry
☐	☒ MRI-based neuroimaging

Animals and other research organisms

Policy information about studies involving animals; ARRIVE guidelines recommended for reporting animal research, and Sex and Gender in Research

Laboratory animals	A total of 10 female wild-type C57BL/6J mice (Mus musculus), aged 8–12 weeks, were used in this study. The mice were obtained from The Jackson Laboratory, strain #000664, and were not genetically modified. Seven animals were included in the primary experimental cohort and formed the basis of the reported imaging and lesion analysis. An additional three animals were used during manuscript revision to optimize imaging parameters and acquire improved representative images, as requested during peer review. Data from these additional animals were used for illustrative and methodological purposes and did not alter the study design or overall conclusions. A single adult female White Alpine Sheep (Ovis aries), aged 3 years and 7 months, was included in this study.
Wild animals	This study did not involve wild animals.
Reporting on sex	Only female mice and female sheep were used in this study. The study was designed to evaluate the technical performance and feasibility of the integrated focused ultrasound and optoacoustic imaging system rather than to investigate biological differences between sexes. Consequently, no sex-based comparisons were performed, and the findings should be interpreted as specific to female animals.
Field-collected samples	This study did not involve samples collected from the field.
Ethics oversight	All experiments were performed in accordance with the Swiss Animal Welfare Act and were approved by the Cantonal Veterinary Office Zürich.

Note that full information on the approval of the study protocol must also be provided in the manuscript.

Plants

Seed stocks	No plant material was used in this study.
Novel plant genotypes	No plant material was used in this study.
Authentication	No plant material was used in this study.

Magnetic resonance imaging

Experimental design

Design type	Structural MRI was used for anatomical assessment and post-treatment lesion validation. No task-based or resting-state functional MRI was performed. Mouse MRI was acquired after FUS treatment. The sheep underwent baseline anatomical MRI for morphological assessment and neuronavigation, followed by post-treatment MRI for lesion assessment.

Design specifications	Mouse experiments involved one post-treatment MRI session per imaged animal. The principal lesion analysis comprised 3 mice and 7 sonication points. The sheep underwent one baseline MRI session, performed 30 min after anesthetic induction, and one post-treatment MRI session. The post-treatment protocol repeated the baseline sequences and included additional lesion-sensitive sequences.
Behavioral performance measures	Not applicable. Only structural MRI was performed, and no behavioral tasks or performance measures were collected.

Acquisition

Imaging type(s)	Structural MRI: T1-weighted, T2-weighted, proton-density-weighted and FLAIR imaging. No functional, diffusion or perfusion MRI was performed.
Field strength	Mouse: 7 T. Sheep: 3 T.
Sequence & imaging parameters	Mouse MRI: Images were acquired using a horizontal-bore 7 T small-animal MRI scanner (PharmaScan, Bruker BioSpin, Germany), equipped with a CryoProbe and operated using ParaVision 6.0.1. Basic frequency, reference power and shimming adjustments were performed before acquisition. A high-resolution 2D RARE sequence was used to acquire 16 sagittal slices with the following parameters: repetition time (TR), 4000 ms; echo time (TE), 11.2337 ms; field of view (FOV), 16 × 16 mm²; matrix size, 267 × 267; in-plane resolution, 60 × 60 µm²; slice thickness, 0.4 mm; slice gap, 0 mm; RARE factor, 6; and number of averages, 8. A second high-resolution 2D RARE sequence was used to acquire 20 coronal slices with the following parameters: TR, 4000 ms; TE, 59.84 ms; echo spacing, 19.946 ms; bandwidth, 32,894.7 Hz; FOV, 10 × 12.7 mm²; matrix size, 400 × 512; in-plane resolution, 25 × 25 µm²; slice thickness, 0.4 mm; slice gap, 0.1 mm; RARE factor, 8; and number of averages, 16.<br><br>Sheep MRI: Images were acquired using a 3 T clinical MRI scanner (Philips Elition 3.0T X, Philips AG, Zurich, Switzerland) equipped with a 32-channel head coil (dStream HeadSpine solution, Philips AG). The animal was positioned head-first in a supine orientation. Baseline anatomical imaging comprised a 3D T1-weighted turbo field echo (TFE) sequence with TR, 10 ms; TE, 4.6 ms; flip angle, 8°; FOV, 210 × 210 × 176 mm³; isotropic voxel size, 0.8 mm; and acquisition time, 6 min 7 s. A 3D T2-weighted turbo spin echo (TSE) sequence was also acquired with TR, 2500 ms; TE, 300 ms; flip angle, 90°; FOV, 250 × 250 × 174 mm³; isotropic voxel size, 0.8 mm; and acquisition time, 6 min 40 s.<br><br>Following treatment, the baseline protocol was repeated and supplemented with sequences optimized for lesion detection. A coronal proton-density-weighted turbo spin echo (PDW-TSE) sequence was acquired with TR, 2822 ms; TE, 30 ms; refocusing flip angle, 90°; FOV, 160 × 120 × 171 mm³; voxel size, 0.50 × 0.50 × 3.00 mm³; and acquisition time, 3 min 40 s. A transverse PDW-TSE sequence was acquired with TR, 2500 ms; TE, 30 ms; refocusing flip angle, 90°; FOV, 170 × 170 × 135 mm³; voxel size, 0.40 × 0.43 × 3.00 mm³; and acquisition time, 3 min 15 s. A sagittal PDW-TSE sequence with spectral presaturation with inversion recovery (SPAIR) fat suppression was acquired with TR, 3567 ms; TE, 30 ms; refocusing flip angle, 90°; FOV, 170 × 175 × 152 mm³; voxel size, 0.42 × 0.53 × 3.00 mm³; and acquisition time, 6 min 36 s. A transverse fluid-attenuated inversion recovery (FLAIR) sequence was acquired with TR, 11,000 ms; TE, 125 ms; inversion time, 2800 ms; refocusing angle, 120°; FOV, 200 × 200 × 115 mm³; voxel size, 0.45 × 0.62 × 3.00 mm³; and acquisition time, 4 min 46 s. Post-treatment MRI was performed following intravenous administration of the gadolinium-based contrast agent DOTAREM at 0.2 mL kg^{-1} (0.1 mmol kg^{-1}).
Area of acquisition	Whole-brain MRI acquisition was performed in both the mouse and sheep experiments. The field of view was selected to cover the entire brain.

Diffusion MRI ☐ Used ☒ Not used

Preprocessing

Preprocessing software	Mouse data were acquired using ParaVision 6.0.1 and processed in AFNI. The AFNI “to3d” command was used to convert Bruker data to NIfTI format; AFNI was then used for visualization and TIFF export. Sheep data were analysed and visualized using 3D Slicer [Version 5.10]. Manual feature-based OA–MRI registration was performed using custom scripts in MATLAB R2023a through geometric transformation, rotation and rescaling.
Normalization	No spatial normalization to a standardized atlas was performed. MRI data remained in individual subject space.
Normalization template	Not applicable. No standardized anatomical template was used.
Noise and artifact removal	Basic frequency, reference power and shimming adjustments were performed before mouse MRI acquisition. No post-acquisition physiological-noise regression or dedicated denoising procedure was applied. Contrast enhancement was used for visualization of Fig. 3f and Fig. 5c only.
Volume censoring	Not applicable to these structural MRI acquisitions. No MRI volumes or datasets were excluded.

Statistical modeling & inference

Model type and settings	Not applicable. MRI was used for structural visualization, anatomical registration and qualitative validation of treatment-induced lesions. No voxel-wise or group-level statistical model was fitted.
Effect(s) tested	Not applicable. No MRI-based inferential hypothesis testing was performed.

Specify type of analysis: ☐ Whole brain ☒ ROI-based ☐ Both

Anatomical location(s): Analyses focused on the FUS sonication points and contralateral control regions in mice and the targeted sonication region in the sheep. Locations were determined from treatment coordinates, MRI contrast alterations and anatomical landmarks; no automated atlas-labelling algorithm was used.

Statistic type for inference (See Eklund et al. 2016): Not applicable. No voxel-wise or cluster-wise statistical inference was performed.

Correction: Not applicable. No multiple-comparison testing was performed.

Models & analysis

n/a	Involved in the study
☒	☐ Functional and/or effective connectivity
☒	☐ Graph analysis
☒	☐ Multivariate modeling or predictive analysis
