# Supplementary material for: Integrated device for optoacoustically-guided ultrasound surgery
Source: Nat Commun. 2026 Jul 21;17:8871. doi: 10.1038/s41467-026-75692-4 (PMC13500822; doi:10.1038/s41467-026-75692-4)
Supplement: Supplementary file 4 [file 41467_2026_75692_MOESM5_ESM.pdf]

nature portfolio

Peer Review File

Integrated device for optoacoustically-guided ultrasound surgery

Corresponding Author: Dr Hector Estrada

Parts of this Peer Review File have been redacted as indicated to remove third-party material.

This file contains all reviewer reports in order by version, followed by all author rebuttals in order by version.

Version 0:

Reviewer comments:

Reviewer #1

(Remarks to the Author)
This work on Optoacoustic guided high intensity focused ultrasound (OAgFUS) is of great interest for biomedical imaging, medical diagnosis, and treatment. Its novelty and innovative character give it great potential for future medical applications in the field of therapy and monitoring. The work has been carried out and presented in a scientifically sound manner. The results have been verified by MR imaging and histological examinations. I recommend publication in Nature Communication after minor revision, provided that the following issues are clarified/improved:

(1) How efficient is HIFU generation with the hemispherical US array compared to the commonly used focused HIFU transducers? In particular, information regarding achievable US intensity in the focus area, heating power, etc. should be added to the manuscript.

(2) What is the maximum penetration depth for this procedure, given that PA imaging has limitations in terms of penetration depth? Please comment/discuss this point in comparison with USgHIFU and MRgHIFU therapy.

(3) Why is there a slight difference between the xz- and yz-MIP images at HIFU focus (Fig.2A) and the spatial resolution of the OA (Fig.2C)? I would expect these to be exactly the same.

(4) Page 3, chapter 2.2: It is stated "..... hypointense areas at the SPs (solid colored arrows in Fig. 3F), with respect to the CPs (outlined colored arrows in Fig. 3F) in T2-weighted images.". However, there are no arrows in Figure 3F. This probably refers to the dotted circles.

(5) The manuscript often lacks spaces between words, such as in "Modalitythat, suited for, heatingwith". Please check the manuscript carefully in this context.

Reviewer #2

(Remarks to the Author)
The authors present an integrated platform utilizing the same spherical ultrasound transducer for both HIFU stimulation and optoacoustic imaging. They demonstrate ex vivo experiments in the mouse brain and quantify optoacoustic signal changes associated with thermal lesion formation. While the technical integration is clearly described, the clinical significance of this work remains limited, and the novelty appears incremental. Therefore, I do not consider this manuscript suitable for publication in Nature Communications.

1. The overall image quality is poor, making it difficult to derive meaningful biological or clinical significance from the presented data.
2. The presented results do not convincingly support the need for this integrated system. While HIFU is commonly used for tumor ablation, the current study does not demonstrate results relevant to this clinical application.
3. This study was limited to in vitro small animal brain experiments, significantly limiting its clinical significance. To

demonstrate potential clinical significance, in vivo validation using a large animal model is expected to be necessary.

Reviewer #3

(Remarks to the Author)
In this manuscript, authors present a method for high resolution optoacoustic imaging of thermal lesions made with an ultrasound array. It is the same array that transmits for the ultrasound ablation and receives for the imaging. This would allow for precise thermal lesioning in mice for scientific study. The OA signal changes at some point in the temperature rise, presumably when there is thermal coagulation. There are some questions that authors could clarify. I would suggest that authors collect some more images with better quality. In addition, a compelling story on why this level of resolution is needed and how it could be used would be useful. Some specific points are —

Is the OA signal quantitative with temperature or do authors think that they can be quantitative? If not, then it would be better to rewrite the introduction to clarify that while MRI is more expensive, it offers quantitative thermometry.

How you get to an imaging resolution of 780 nm needs some explanation. In addition, there are no OA images presented. I would like to see this 780 nm resolution. How is all the data handled? Across a mouse brain of 10 mm x 5 mm x 5 mm, this is 10^12 pixels. Presumably, the imaging is done over a much smaller FOV. But all this needs clarity.

It is not clear in Fig. 3 Why the inner VOI doesn't have as high a signal change as the outer VOIs. This needs explanation. To first order, no matter where the VOI is spatially, coagulation will occur at some temperature, the OA signal is the same as a function of temperature, so the coagulation should be at the same OA level. It makes no sense that the OA signal is higher in the outer VOIs. Maybe there is something about the way these signals are normalized, but this needs clarity.

The Figure 3 T2s are motion corrupted. It's really hard to see the lesion with this level of artifact.

Version 1:

Reviewer comments:

Reviewer #1

(Remarks to the Author)
This manuscript has been thoroughly revised in response to the reviewers comments, resulting in an improvement in quality that meets the standards of Nature Communications. In particular, the objections regarding clinical relevance and novelty were addressed by conducting additional experiments as part of an in vivo animal study on sheep. In addition, new MR images were taken with better image quality, from which the clinically relevant information is now clearly visible.
In general, the presented work on Optoacoustic guided high intensity focused ultrasound is of great interest for the biomedical research community in terms of diagnosis and treatment. Furthermore, the work has been carried out in a technically sound manner, and the data supports the conclusions. Which brings me to the decision to recommend the work for publication in Nature Communication.

Reviewer #2

(Remarks to the Author)
General Comments
The "Integrated device for optoacoustically-guided ultrasound surgery" proposed an interesting approach of integrating real-time optoacoustic tomography with concurrent focused ultrasound delivery using a single high-frequency spherical transducer array. Developing such an integrated system and demonstrating its feasibility in both mouse and sheep models is highly significant for the field of image-guided therapy. While the potential of the fusion system is impressive, I believe that major revisions are required to ensure the manuscript has the potential to meet the high standards of the journal.

Comment 1
Specifically, the advantages of the OAgFUS system should be clearly demonstrated through two main aspects: (1) OA structural-based target navigation during FUS and (2) simultaneous OA-based lesion monitoring. The current presentation of the sheep data in Figure 5 could be further strengthened to fully support the "OA-guided" aspect of the OAgFUS system. Although the authors mention that OA imaging offered valuable structural information to avoid vasculature during the procedure, the manuscript would benefit from the inclusion of representative OA images of the sheep brain, similar to those provided for the mouse model. Additionally, the current results (in sheep model) seem to have some limitations in fully demonstrating the significant clinical potential of the OAgFUS system. Given that the sheep data appears to be limited to a single specimen, merely showing the changes in OA signals from VOI1 to VOI3 does not sufficiently showcase this potential and further direction. I suggest that the authors consider adding further analysis based on this result, such as a more detailed assessment of the lesion dimensions in comparison with the histological findings. Overall, I suggest supplementing the results to more clearly demonstrate the significant advantages inherent to the OAgFUS system.

Comment 2
There is a discrepancy in figure referencing within the Methods section (in 'Quantitative analysis of lesion dimension' section). The authors refer to "Fig. 3b bottom row" as showing the OA signal's exponential increase for "mere heating". But it

seems that there are missing. Additionally, the authors stated that a high R2 from exponential fitting was assumed to indicate a thermal lesion, but wouldn't a high R2 actually be indicative of "mere heating" instead? Please check this point.

Comment 3
The current methodology for predicting lesion size based on OA signals relies on several assumptions, making it difficult to clearly interpret the accuracy of the results, while authors claim that the OA-derived VLrange closely approximates the histological findings. However, this claim appears somewhat vague, as it is based on the average histological volume falling within the relatively wide, predefined ranges of VOI2 or VOI3. To provide a more persuasive validation, the authors should consider to examine the feasibility of providing a one-to-one correspondence between the OA-estimated range and the individual histological measurement for each of the seven lesions. Additionally, the authors should consider including a representative figure that displays the specific OA signal traces alongside the corresponding histological lesion size for a single case to better illustrate the correlation between the two modalities. Overall, please consider providing sufficient justification to demonstrate the rigor of the methodology used for estimating lesion size.

Comment 4
The current estimation of lesion volume appears to be conducted within a very broad range. Please providing a more detailed discussion on the clinical significance of this approach in the aspect of further directions, along with strategies or directions for achieving more precise lesion evaluation, would help convince readers of the potential demonstrated in this study.

Comment 5
What does the standard deviation (shaded area) in Fig 3d stand for? Please clarify this.

Comment 6
In Fig 5b, please also display the VOI information to ensure consistency and clarity.

Reviewer #3

(Remarks to the Author)
The manuscript is much improved.

Thank you for the clarification that 780 nm is the laser wavelength. It would be helpful to specify this in the caption to Fig. S1.

Fig. S2. is somewhat incomprehensible and would benefit from clarification.

Version 2:

Reviewer comments:

Reviewer #2

(Remarks to the Author)
The authors have adequately addressed the concerns raised in the previous review. The inclusion of the large-animal OA images and the 1:1 lesion comparison data has clarified the system's capabilities and the validity of the methodology. Additionally, the limitations of the proposed approach are now properly discussed in the manuscript. I have no further comments and recommend the manuscript for publication.

Open Access This Peer Review File is licensed under a Creative Commons Attribution 4.0 International License, which permits use, sharing, adaptation, distribution and reproduction in any medium or format, as long as you give appropriate credit to the original author(s) and the source, provide a link to the Creative Commons license, and indicate if changes were made.
In cases where reviewers are anonymous, credit should be given to 'Anonymous Referee' and the source.
The images or other third party material in this Peer Review File are included in the article's Creative Commons license, unless indicated otherwise in a credit line to the material. If material is not included in the article's Creative Commons license and your intended use is not permitted by statutory regulation or exceeds the permitted use, you will need to obtain permission directly from the copyright holder.
To view a copy of this license, visit https://creativecommons.org/licenses/by/4.0/

Point to point response to the Reviewer's comments

Reviewer #1 (Remarks to the Author):

This work on Optoacoustic guided high intensity focused ultrasound (OAgFUS) is of great interest for biomedical imaging, medical diagnosis, and treatment. Its novelty and innovative character give it great potential for future medical applications in the field of therapy and monitoring. The work has been carried out and presented in a scientifically sound manner. The results have been verified by MR imaging and histological examinations. I recommend publication in Nature Communication after minor revision, provided that the following issues are clarified/improved:

Reply: We thank the Reviewer for their time and comments. We hope they find the changes to the manuscript satisfactory.

(1) How efficient is HIFU generation with the hemispherical US array compared to the commonly used focused HIFU transducers? In particular, information regarding achievable US intensity in the focus area, heating power, etc. should be added to the manuscript.

Reply: From a physics standpoint, the array exhibits an efficiency equivalent to that of a single-element transducer, provided that both the angular coverage and the frequency spectrum are comparable and the same piezoelectric material is used. From a fabrication standpoint, the array has a slightly smaller radiating area because its elements must be cut and electrically isolated. In our design, however, the elements are quadrilateral, so the area lost during dicing is minimal and does not significantly affect efficiency.
In a real world scenario, both the angular coverage and the frequency spectrum of our array differ substantially from those of conventional HIFU single-element transducers (and arrays). First, clinical HIFU systems typically employ reduced angular coverage to reach deeper focal targets. Second, our array requires a wide bandwidth to enable imaging, whereas conventional HIFU transducers use narrow bandwidths to maximize efficiency.
Ultimately, the efficiency reduction associated with the wide bandwidth of our array is offset by its large angular coverage. We added the following details to "**Methods: System characterization**":
"We measured the array's pressure using a calibrated hydrophone (Precision Acoustics, UK) in a degassed water tank. To avoid damaging the hydrophone, we measured its focus at low pressure and then measured the pressure out of focus increasing the voltage to extrapolate the peak pressure. Under these operating conditions, the array achieves a peak pressure of 6.8 MPa when driven at ±6.5 V and 5 MHz, corresponding to a spatial peak time average intensity (I_{SPTA}) of 716 W/cm² at a 49 % duty cycle and a mechanical index MI = 3. The peak heating power density, assuming an attenuation coefficient of α = 0.92 cm^{-1} (α_0 = 0.8 dB/(cm·MHz) for brain tissue)[61], is calculated to be 1.3 kW/cm³."

(2) What is the maximum penetration depth for this procedure, given that PA imaging has limitations in terms of penetration depth? Please comment/discuss this point in comparison with USgHIFU and MRgHIFU therapy.

Reply: Our integrated OAgFUS system is fundamentally designed for applications where high resolution and vascular contrast are the primary requirements, as it is inherently limited by light attenuation, achieving a penetration depth of approximately 1-2 cm in soft tissue. Consequently, this makes our platform ideally suited for clinical use in superficial or minimally invasive scenarios, rather than for deep-tissue applications. To clarify these aspects:

- We have briefly revised the **Introduction** to incorporate USgHIFU as terminology, and added two references to back up the discussion about this technology:
  "Ultrasound-imaging-guided HIFU (USgHIFU), with comparatively lower hardware costs, can provide real-time monitoring with high spatial resolution, together with a penetration depth >10 cm.[11, 12]"

- We have revised the **Discussion** to explicitly discuss the penetration depth, and compare it to USgHIFU and MRgHIFU:
  "While the exceptional resolution and label-free vascular sensitivity of our integrated OAgFUS system enable precise targeting and monitoring of thermal lesion formation, its effective penetration depth remains confined to approximately 1-2 cm due to the strong light attenuation.[55] In contrast to the deeper reach of USgHIFU (>10 cm) or full-body MRgHIFU,[10,56] our system has potential for broader therapeutic applications, including the treatment of shallow liver tumors, subcutaneous lesions, and other localized pathologies where precise and controlled thermal ablation is required."

(3) Why is there a slight difference between the xz- and yz-MIP images at HIFU focus (Fig.2A) and the spatial resolution of the OA (Fig.2C)? I would expect these to be exactly the same.

Reply: The difference between both maps arises because the FUS focus (**Fig. 2a**) captures the *transmit-side* response of the system, which involves generating waves at a specific frequency (*i.e.*, 5 MHz). In contrast, the OA point-spread-function (now **Fig. 2b**) represents the detection of wideband laser-generated waves. In order to clarify these aspects:

- We have remodeled **Fig. 2**, thus making it more compact, while keeping the same amount of information:

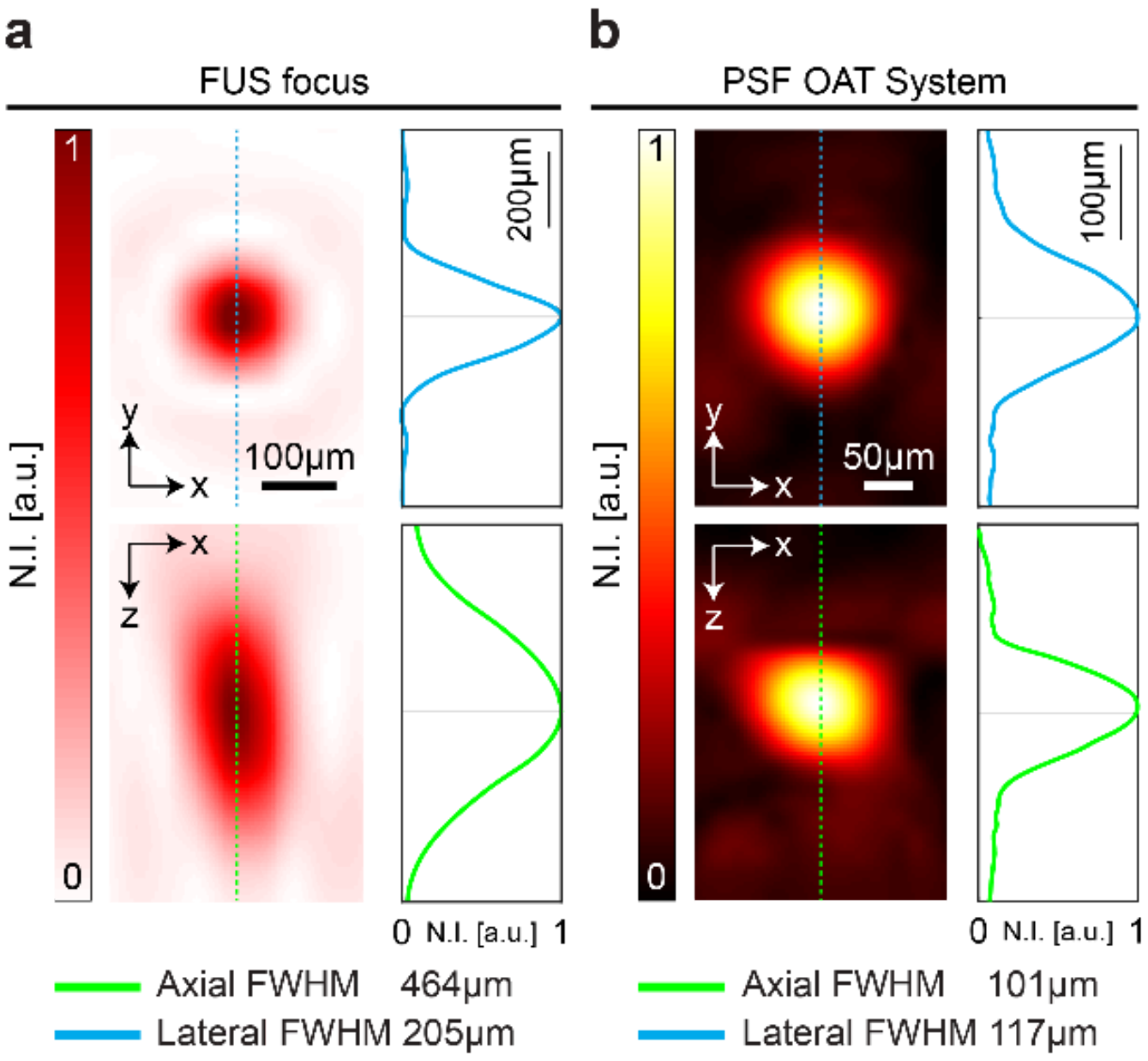

- The colormaps in **Fig. 2a** and **Fig. 2b** (old Fig. 2c) are now different, thus providing the reader a visual distinction between both measurements, emphasizing that they represent different system properties.

- The caption for **Fig. 2b** (old Fig. 2C) has been extended to explicitly state that the panel depicts the PSF of the OA system.

- The label in **Fig. 2b** (old Fig. 2c) reading "OA spatial resolution" has been replaced with "PSF OAT System".

- In "**Methods: System characterization**", the text has been revised to further clarify the distinction between these two quantifications:
  "We measured the array's pressure using a calibrated hydrophone (Precision Acoustics, UK) in a degassed water tank. To avoid damaging the hydrophone, we measured its focus at low pressure and then measured the pressure out of focus, increasing the voltage to extrapolate the peak pressure. Under these operating conditions, the array achieves a peak pressure of 6.8 MPa when driven at ±6.5 V and 5 MHz, corresponding to a spatial peak time average intensity (I_{SPTA}) of 716 W/cm² at a 49% duty cycle and a mechanical index MI = 3. The peak heating power density, assuming an attenuation coefficient of $\alpha = 0.92\,cm^{-1}$ ($\alpha_0 = 0.8$ dB/(cm·MHz) for brain tissue), is calculated to be 1.3 kW/cm³.
  The US focus at 5 MHz is elliptical (**Fig. 2a**). The spatial extent of the pressure field, quantified by its full width at half maximum (FWHM), is 464 µm axially and 205 µm laterally. We calculated the ellipticity of the focus (*f*) as follows:

$$f = (FWHM_{axial} - FWHM_{lateral}) / FWHM_{axial}$$

  which results in a value of f = 0.56. To characterize the system's OA imaging spatial resolution, the point spread function (PSF) was measured by imaging a single ≈ 30 µm microsphere (**Fig. 2b**). This yielded axial and lateral resolutions of 101 µm and 117 µm, respectively, as defined by the FWHM."

(4) Page 3, chapter 2.2: It is stated "..... hypointense areas at the SPs (solid colored arrows in Fig. 3F), with respect to the CPs (outlined colored arrows in Fig. 3F) in T2-weighted images.". However, there are no arrows in Figure 3F. This probably refers to the dotted circles.

Reply: We have taken the following measures to clarify this aspect:

- We have updated **Fig. 3f** with a new set of MR images to improve visualization of the thermal lesions:

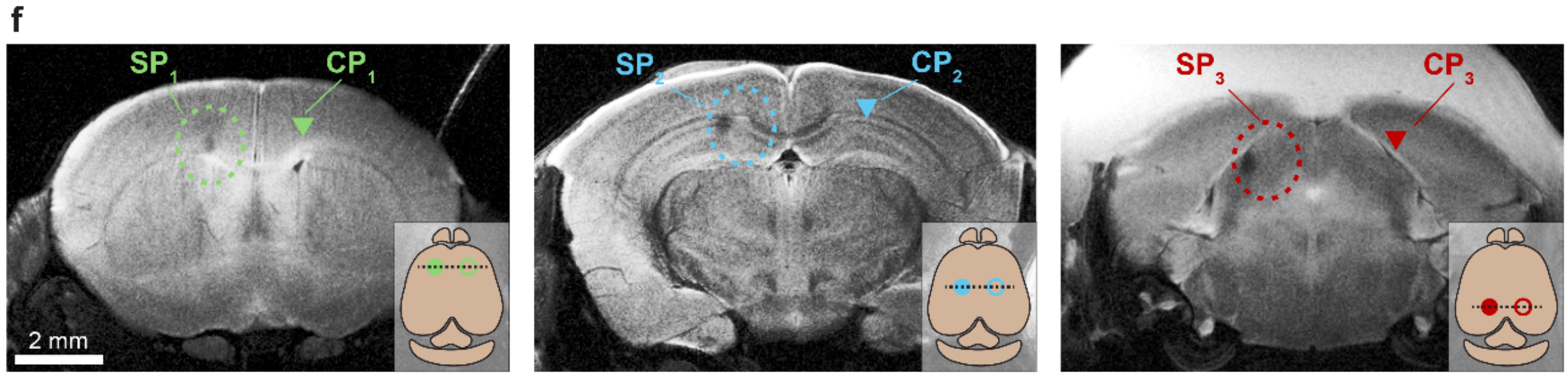

- In **Fig. 3f**, we have highlighted the sonication points (SP_1, SP_2 and SP_3) using a dotted-line contour and the control points (CP_1, CP_2 and CP_3) using solid-colored arrows.

- Updated the caption for **Fig. 3f**, specifying how we highlight the sonication and control points:
  "T2 MRI coronal scans performed after sonication reveal regions with different contrast, comparing SPs regions (left hemisphere, delineated by dotted-line contours) to CPs regions (right hemisphere, pointed by solid colored arrows). Image contrast is enhanced to highlight the dark region in the SPs."

- Updated "**Results: OA monitoring of HIFU-induced lesions**" to correctly refer to the sonication and control points:
  "Following FUS treatment, we imaged the mouse head in a 7T MRI scanner to assess tissue alterations (**Methods**). This preliminary assessment revealed hypointense areas at the SPs (dotted-line contours in **Fig. 3f**), with respect to the CPs (solid colored arrows in **Fig. 3f**) in T2-weighted images."

(5) The manuscript often lacks spaces between words, such as in "Modality that, suited for, heatingwith". Please check the manuscript carefully in this context.

Reply: We have carefully reviewed the manuscript and corrected the missing spaces between words throughout the text, as well as any other spelling mistakes.

Reviewer #2 (Remarks to the Author):

The authors present an integrated platform utilizing the same spherical ultrasound transducer for both HIFU stimulation and optoacoustic imaging. They demonstrate ex vivo experiments in the mouse brain and quantify optoacoustic signal changes associated with thermal lesion formation. While the technical integration is clearly described, the clinical significance of this work remains limited, and the novelty appears incremental. Therefore, I do not consider this manuscript suitable for publication in Nature Communications.

Reply: We thank the Reviewer for their time and comments and hope they find the substantial changes to the manuscript satisfactory. We have successfully performed in vivo experiments in a large animal model (i.e., a sheep), demonstrating the translatability of our method from a mouse brain ex vivo proof-of-concept to a living brain that mimics human anatomical and physiological scales more closely. This significant advancement directly addresses the limitation noted, as it: (1) proves the operational feasibility and robustness of our dual-mode transducer in a demanding in vivo setting, (2) establishes the potential for monitoring HIFU thermal lesion formation in real-time within a deep, large tissue volume, and (3) substantiates the clear clinical pathway of our technology for precise, image-guided therapeutic interventions. We believe this major new experimental component elevates the impact and novelty of our work beyond an incremental integration, providing a pivotal translational step that strongly aligns with the scope of Nature Communications. The new results have been fully integrated into the revised manuscript, and we are confident they address the Reviewer's concerns.

1. The overall image quality is poor, making it difficult to derive meaningful biological or clinical significance from the presented data.

Reply: The issues observed in the previous manuscript were caused by susceptibility artifacts at the brain edges during MRI acquisition. In the revised manuscript, we conducted new sets of experiments and further optimized the MRI scanning sequence to improve image quality and achieve higher spatial resolution. Accordingly, we have implemented the following modifications in the manuscript:

- We have updated **Fig. 3f** with a new set of MR images to improve visualization of the thermal lesions:

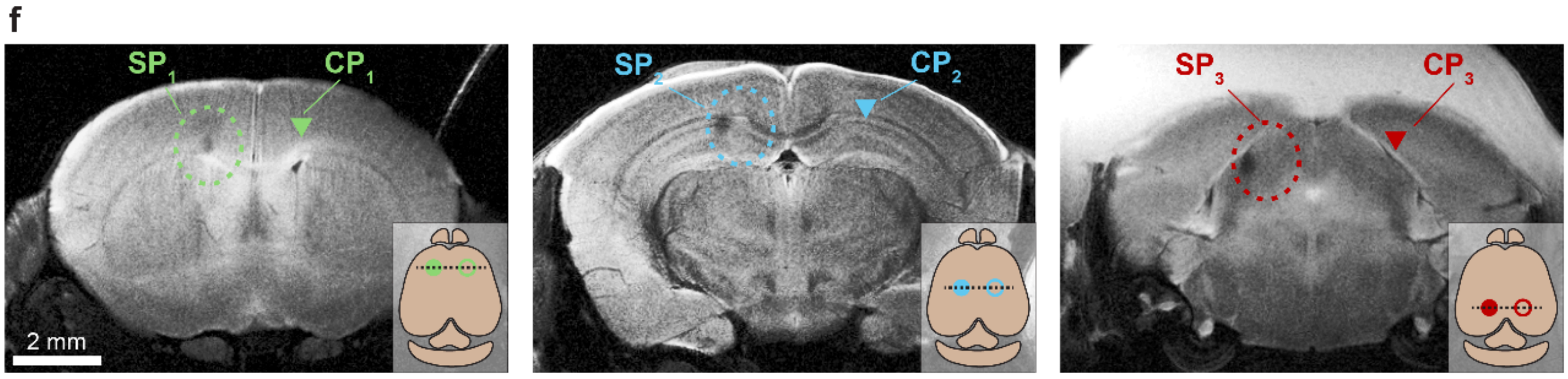

- We have expanded section "**Methods: MRI scanning and image processing**" with the corresponding acquisition parameters, related to the new set of MR images: "MRI was used to validate the coagulated regions in the mice and sheep brains. In the mouse model, all MRI images for post-FUS validation were acquired by a horizontal bore small animal 7T MRI scanner (PharmaScan, Bruker BioSpin, Germany) using a CryoProbe with the software ParaVision 6.0.1. Adjustments to the

basic frequency, reference power, shimming were performed before the data acquisition. We used a high resolution 2D RARE sequence to acquire 16 sagittal slices, with the following parameters: repetition time of 4000 ms, echo time of 11.2337 ms, FOV of 16 mm × 16 mm, matrix size of 267 × 267, resolution of 60 μm × 60 μm, slice thickness of 0.4 mm, slice gap of 0 mm, RARE factor of 6, average of 8. Subsequently, we used a 2D RARE sequence to acquire 20 coronal slices with high resolution using the following parameters: repetition time of 4000 ms, echo time of 59.84 ms, echo spacing of 19.946 ms, bandwidth of 32894.7 Hz, FOV of 10 mm × 12.7 mm, matrix size of 400 × 512, resolution of 25 μm × 25 μm, slice thickness of 0.4 mm, slice gap of 0.1 mm, RARE factor of 8, and average of 16."

- MR sagittal images originally presented in Fig. 4 have been post-processed using ImageJ, by applying brightness/contrast adjustments and are now available as **Supplementary Fig. 2**:

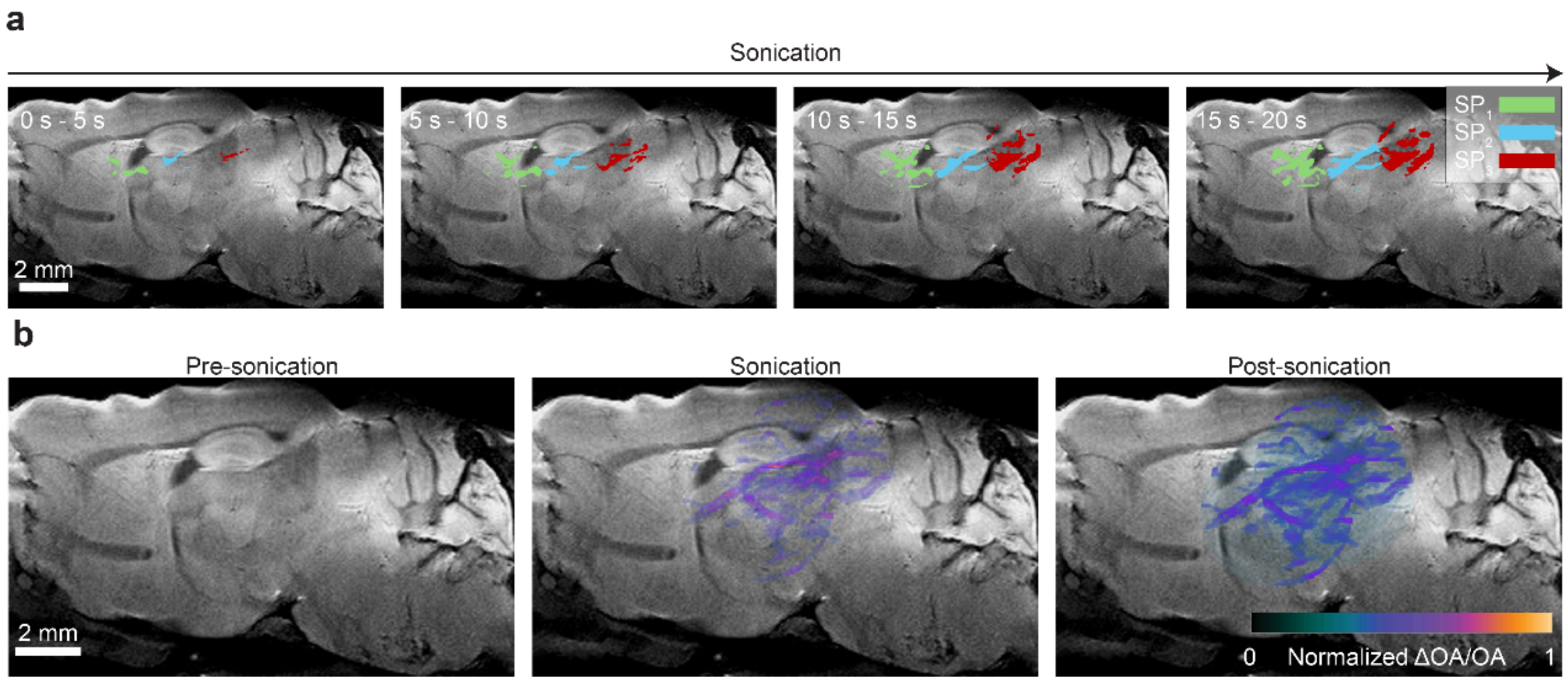

2. The presented results do not convincingly support the need for this integrated system. While HIFU is commonly used for tumor ablation, the current study does not demonstrate results relevant to this clinical application.

Reply: The primary objective of this work is to propose a novel integrated system capable of real-time, high-precision thermal monitoring during focused ultrasound therapy, addressing a recognized technological limitation in the field. Although clinical HIFU tumor-ablation systems already exist, none currently provide true 3D imaging within the same compact form factor and frame rate fast enough to monitor thermal therapy. While our prototype is still at an early-stage to claim clinical utility, it demonstrates a viable alternative pathway to existing solutions, which typically depend on bulky and costly MRI guidance (low temporal resolution, typically 1-3 Hz), rely on limited 2D ultrasound imaging, or use optical methods that cannot penetrate beyond approximately 1 mm in depth.
To further strengthen the clinical translatability of our system, we made a large effort to include an in vivo validation in a large animal model (detailed in our response to Question 3). These new results successfully demonstrate that our integrated system can be translated to more clinically relevant scenarios.

3. This study was limited to in vitro small animal brain experiments, significantly limiting its clinical significance. To demonstrate potential clinical significance, in vivo validation using a large animal model is expected to be necessary.

Reply: We agree that in vivo validation in a large animal model is essential to demonstrate the translatability of our device. Therefore, we have performed an in vivo experiment in a large animal (i.e., sheep) model. The new results demonstrate the robustness of our method for real-time optoacoustic monitoring of HIFU thermal lesion formation at clinically relevant depths and scales, thereby directly addressing the Reviewer's concern. We have taken the following actions in the manuscript to include the in vivo validation:

- We have adapted the **Abstract** of the paper to highlight the relevance of this result and put it in context within the entire work:
  "We evaluated the performance of the system in the mouse brain ex vivo and further assessed its clinical translability in a living sheep, showing that we could induce a confined thermal lesion beneath the cortical surface, as confirmed by magnetic resonance imaging and histology."

- We have extended the last paragraph of the **Introduction** to include the new in vivo data:
  "The system's performance is demonstrated in vivo in a large-animal model, underscoring its potential for translational image-guided ultrasound research."

- We have added a new section ("**Result: In vivo validation in large animal model**") to explain the results obtained from this newly added study:
  "We conducted in vivo validation in a craniotomized sheep model under anesthesia to evaluate the intraoperative translational potential of OAgFUS (**Fig. 5a**, left). The transducer was adapted for a larger-scale brain while keeping all other setup elements and parameters unchanged. Specifically, elements with a lower central frequency were used to increase the imaging penetration depth (**Methods**).
  We defined a SP 1 cm below the cortical surface (**Fig. 5a**, right), located in the mesial temporal-diencephalic junction, involving adjacent hippocampal, thalamic/hypothalamic, and piriform lobe structures. We computed the mean ΔOA/OA for the different VOIs, using larger values of R_1 and R_2 to account for the larger focus (**Fig. 5b**, **Fig. 3b**, and **Methods**). In the resulting traces, ΔOA/OA for all VOIs starts to increase due to the temperature rise from the FUS-mediated thermal energy deposition. Afterward, VOI_1 undergoes a marked trend change approximately 12 s into sonication, while VOI_2 plateaus until FUS is turned off. VOI_3 shows a minor correction but maintains a positive trend. These results suggest that the bulk of the thermal lesion is represented by VOI_1 which would correspond to a maximum lesion size of 1 mm wide and ~ 2.3 mm long.
  After FUS, we performed a postoperative MR (**Fig. 5c**, **Methods**). The assessment of T2-weighted MRI reveals a clearly distinguishable area in the target SP, characterized by a hyperintense area with central hypointensity. The lesioned area was evaluated in formalin-fixed paraffin-embedded brain tissue sections. In the H&E-stained sections, rarefication of the cerebral parenchyma was observed in the white matter of the cerebrum. In addition to tissue ruptures, small accumulations of pale eosinophilic fluid were present. Increased cellularity in terms of gliosis or inflammatory cell infiltration was not detected (**Fig. 5d**, **Methods**)."

- We have elaborated a new figure that illustrates the experimental setup and the obtained results together with the evidence to prove those. To accommodate this major revision, we have rearranged the distribution of figures:
  - New **Fig. 4** is old Figure 5.
  - Old Figure 4 has been moved to **Supplementary Figure 2**.
  - We have elaborated a new figure (**Fig. 5**) related to the newly added in vivo sheep experiments:

[REDACTED]

- We have edited the introduction of the **Discussion** to include these new results:
  "We have demonstrated the functionality of our integrated device, capable of real-time OA imaging concurrently with FUS sonication, and proven its performance in an in vivo animal model. [...] Using real-time OA imaging, we navigated different animal models' brains and defined target volumes for FUS application."

- We have extended the interpretation of the MR scans in the **Discussion**:
  "Using the MRI scans, we were able to identify tissue alterations in regions corresponding to the SPs, seen as hypointense areas. In the mouse model, the strength of this reproducibility is further supported by MRI from multiple specimens, each sonicated in different regions along the anterior-posterior axis, all of which showed consistent lesion size and depth. These findings align with previous reports where FUS-treated regions were similarly presented as hypointense zones on T2-weighted MRI scans.[48] However, in the sheep model, the hypointense alteration is enveloped by a hypertintense tissue. The latter may be due to vacuolization surrounding the lesion, probably caused by edema."

- We have extended the histological analysis in **Discussion**:
  "Regarding the in vivo sheep brain histology, the H&E-stained sections revealed mechanical disruption of the white matter, characterized by parenchymal loosening and tissue rupture, in the absence of a discernible gliotic or inflammatory response."

- We have revised the text related to key aspects to consider for the in vivo application in **Discussion**:
  "Aiming for an in vivo application of our integrated device, we have tackled several key aspects, such as the potential for blood vessel rupture, and the influence of nearby vasculature on tissue heating. Preventing blood vessel rupture is important to avoid internal hemorrhages and other complications.[51,52] On the other hand, blood vessels act as heat sinks, dissipating thermal energy and leading to uneven thermal distribution, which in turn affect the size and shape of thermal lesions.[53] The proximity, size, and blood flow rate play crucial roles in determining the extent of this effect.[54-56] This challenge is particularly pronounced in brain FUS applications, where precise thermal control is essential to avoid unintended damage to healthy surrounding tissues. Consequently, accounting for vascular cooling effects during FUS planning and execution is vital, especially in highly vascularized regions like the brain. We demonstrated in vivo lesioning capabilities well below the cortical surface (~ 1cm), where OA imaging offered valuable structural information for avoiding vasculature within the focal zone. We were able to induce a highly localized thermal lesion, without disrupting functional vasculature and preserving surrounding tissue. Thus, we have reduced the risk of vascular damage and minimizing interference with critical circulatory pathways.[57]"

- We have revised our last remarks in the **Discussion**:
  "Our system has the potential for inducing thermal lesions in a clinically relevant scenario, although further integration with functionalities like neuronavigation and image fusion with preoperative data needs to be implemented to meet current neurosurgery standards. Our approach enables brain vasculature imaging, FUS delivery, and a quantitative, assessment of thermal lesion formation in the tissue around the focus, that does not rely on temperature estimation. Furthermore, it provides real-time feedback on the lesion size by analyzing the OA signals acquired during sonication. This allows for continuous monitoring of lesion progression, enabling on the fly adjustments to the thermal dose and precise coordination of contiguous SPs to effectively sonicate larger volumes. Future directions of this work include integrating pulse-echo US imaging within the same device, together with Doppler ultrasound, thus providing complementary functional data by assessing both structural visualization and vasculature hemodynamics. Altogether, this technique opens a promising clinical avenue for new translational applications of HIFU treatments."

- We have revised **Methods**, thus incorporating all the new approaches needed for the implementation of this newly added experiment. The modifications are listed below.

  - "**Methods: Optoacoustic guided HIFU system**" has been extended to describe the OAgFUS adapted for the in vivo sheep experiments:
    "The OAgFUS probe was adapted for in vivo sheep experiments (**Fig. 5a**) operating at a central frequency of 4 MHz with >45 % bandwidth, and used for both volumetric OA data acquisition and US therapy."

  - "**Methods: Animal model**" has been extended to describe the sheep as an animal model as well as different aspects regarding animal license and logistics:
    "A single adult female White Alpine Sheep (Ovis aries, WAS breed) was included in this study (animal licence number ZH109/2024). The animal

originated from a Maedi-Visna-free herd, as confirmed by prior serological testing. The source herd was routinely vaccinated against Pasteurella and Clostridia and followed a regular deworming protocol under veterinary supervision. Ongoing health management included a general physical examination every four weeks and hoof trimming every eight weeks.

The sheep was transported in accordance with animal welfare regulations. Following arrival, it underwent a minimum acclimatization period of seven days. A veterinarian then performed a baseline physical examination and recorded its body weight. Only animals confirmed to be healthy, non-pregnant, and displaying normal clinical parameters were enrolled in the study.

Prior to surgery, pregnancy was ruled out using a rapid visual pregnancy test (Idexx Alertys) combined with an ultrasound examination. As part of the pre-anesthetic assessment, a 10–15 mL blood sample was collected from the vena jugularis via a vacutainer system for a complete blood count and serum chemistry panel."

- A new point has been added ("**Methods: Anesthesia protocol**") to detail anesthesia procedures for the two animal models used, describing in details the protocol for the sheep:

"The sheep received a standardized premedication regimen after a 24-hour fast, prior to anesthesia induction. Intramuscular buprenorphine (0.01 mg/kg BW im, 0.03mL/kg) and medetomidine (0.005-0.01 mg/kg BW) were administered 30–45 minutes beforehand. Following intravenous catheter placement, carprofen (4 mg/kg BW, 0.08mL/kg BW) was given for pre-emptive analgesia. Induction was achieved intravenously using midazolam (0.1 mg/kg BW, 0.02mL/kg BW), ketamine (3-5 mg/kg BW, 0.03-0.05 mL/kg BW), and propofol (0.4-4 mg/kg BW, 0.04-0.4 mL/kg BW), titrated to effect. Maintenance of anesthesia employed a balanced protocol of inhaled isoflurane (1–3 %) with oxygen and air (FiO_2 50–70 %), supplemented by intravenous infusions of propofol (0.5-1 mg/kg/h, 0.05-0.1 mL/kg/h) and ketamine (1.2-3 mg/kg/h, 0.012-0.03 mL/kg/h).

After tracheal intubation confirmed by capnography, supportive measures were implemented. These included the application of ophthalmic ointment and continuous intravenous fluid therapy with Ringer's lactate at 5-10 mL/kg/h. Comprehensive physiological monitoring was performed throughout, encompassing electrocardiogram, heart rate, invasive arterial blood pressure, capnography, pulse oximetry, and esophageal temperature. All monitored parameters were recorded at 10-minute intervals."

- A new point has been added ("**Methods: Large animal preparation and integration in the setup**") to detail how we prepared the sheep for the experiment:

"The initial MRI scan, conducted to provide detailed brain morphology, was performed 30 minutes after anesthetic induction (Methods). This was complemented by a CT scan to evaluate cranial thickness and geometry, ensuring procedural safety. Following imaging, the sheep was positioned prone on the surgical table with its limbs secured and its head immobilized within a custom stereotactic frame. A bilateral craniotomy over the frontal and parietal lobes was performed to provide transducer access, exposing the underlying dura mater. All subsequent experimental steps were carried out

prior to dural incision; the dura was then opened bilaterally in a C-shaped pattern using micro-scissors.

For combined OA and FUS procedures, the brain surface was prepared to ensure optimal coupling. A mixture of ultrasound gel and phosphate-buffered saline (PBS) was centrifuged to remove air bubbles and applied to the exposed cortex. A 3D-printed frame surrounded the brain, holding a transparent, degassed water-filled bag, contacting the brain surface. A temperature-controlled silicone coil around the frame maintained the medium at 37 °C to prevent brain cooling. Fine probe position adjustments were made using a multi-axis stage, and real-time OA imaging was used for target visualization and depth selection prior to treatment.

To evaluate the performance of the OAgFUS device, a post-treatment MRI was performed to assess the induced lesions using an intravenous gadolinium-based contrast agent (DOTAREM, 0.2 mL/kg (0.1 mmol/kg)). Following the procedure, the animal was euthanized (Pentobarbital, 150mg/kg bodyweight, 0.5ml/kg, i.v.) Death was confirmed by absence of heartbeat and pupillary reflex. Brain tissue was subsequently harvested for histological analysis to determine the extent of the targeted lesions and to identify any concomitant adverse effects, particularly micro- or macroscopic hemorrhage in off-target regions."

- "**Methods: MRI scanning and image processing**" has been extended, to cover the acquisition details of the sheep MR scans:

"For the sheep study, MRI data were acquired on a 3 Tesla clinical scanner (Philips Elition 3.0T X, Philips AG, Zurich, Switzerland) equipped with a 32-channel head coil (dStream HeadSpine solution, Philips AG). Animals were positioned head-first in a supine orientation. For baseline anatomical evaluation and neuronavigation, two volumetric sequences were performed: a 3D T1-weighted TFE sequence (TR of 10 ms, TE of 4.6 ms, flip angle of 8 °, FOV of 210 mm × 210 mm × 176 mm, isotropic voxel size of 0.8 mm, acquisition time of 06:07 min) and a 3D T2-weighted TSE sequence (TR of 2500 ms, TE of 300 ms, flip angle of 90 °, FOV of 250 mm × 250 mm × 174 mm, isotropic voxel size of 0.8 mm, acquisition time of 06:40 min). Following the therapeutic intervention, the identical pre-treatment protocol was repeated and expanded to include several supplementary sequences optimized for post-treatment lesion detection. These additional sequences consisted of proton density-weighted turbo spin echo (PDW-TSE) scans in coronal (TR of 2822 ms, TE of 30 ms, refocusing flip angle of 90 °, FOV of 160 mm × 120 mm × 171 mm, voxel size of 0.50 mm × 0.50 mm × 3.00 mm, acquisition time of 03:40 min) and transversal (TR of 2500 ms, TE of 30 ms, refocusing flip angle of 90 °, FOV of 170 mm × 170 mm × 135 mm, voxel size of 0.40 mm × 0.43 mm × 3.00 mm; acquisition time of 03:15 min) orientations, a sagittal PDW-TSE sequence with Spectral Presaturation with Inversion Recovery (SPAIR) for fat suppression (TR of 3567 ms, TE of 30 ms, refocusing flip angle of 90 °, FOV of 170 mm × 175 mm × 152 mm, voxel size 0.42 mm × 0.53 mm × 3.00 mm, acquisition time of 06:36 min), and a transversal Fluid Attenuated Inversion Recovery (FLAIR) sequence (TR of 11,000 ms, TE of 125 ms, TI of 2800 ms, refocusing angle of 120 °, FOV of 200 mm × 200 mm × 115 mm, voxel size of 0.45 mm × 0.62 mm × 3.00 mm, acquisition time of 04:46 min). MRI data analysis and visualization were done using 3D Slicer."

- “**Methods: OA signal analysis**” has been extended to specify the dimensions of the volumes of interest (VOIs) used for analyzing the OA signals of the sheep in vivo data:
  “The dimensions of R_1 and R_2 (**Fig. 3B**) used for the OA signal analysis of the in vivo data (**Fig. 5b**) are: 0-1 mm for VOI_1, 1-1.5 mm for VOI_2, and 1.5-2 mm for VOI_3.”

Reviewer #3 (Remarks to the Author):

In this manuscript, authors present a method for high resolution optoacoustic imaging of thermal lesions made with an ultrasound array. It is the same array that transmits for the ultrasound ablation and receives for the imaging. This would allow for precise thermal lesioning in mice for scientific study. The OA signal changes at some point in the temperature rise, presumably when there is thermal coagulation. There are some questions that authors could clarify. I would suggest that authors collect some more images with better quality. In addition, a compelling story on why this level of resolution is needed and how it could be used would be useful. Some specific points are —

Reply: We thank the Reviewer for the thoughtful assessment and valuable suggestions. We agree that enhancing image quality will strengthen the manuscript. We have focused on improving the quality of MR images. The issues observed in the previous manuscript were caused by susceptibility artifacts at the brain edges during MRI acquisition. In the revised manuscript, we conducted new sets of experiments and further optimized the MRI scanning sequence to improve image quality and achieve higher spatial resolution. Accordingly, we have implemented the following modifications in the manuscript:

- We have acquired a new set of MR images from three different mice, included in **Fig. 3f**, with better image quality and better preserved brain morphology:

f

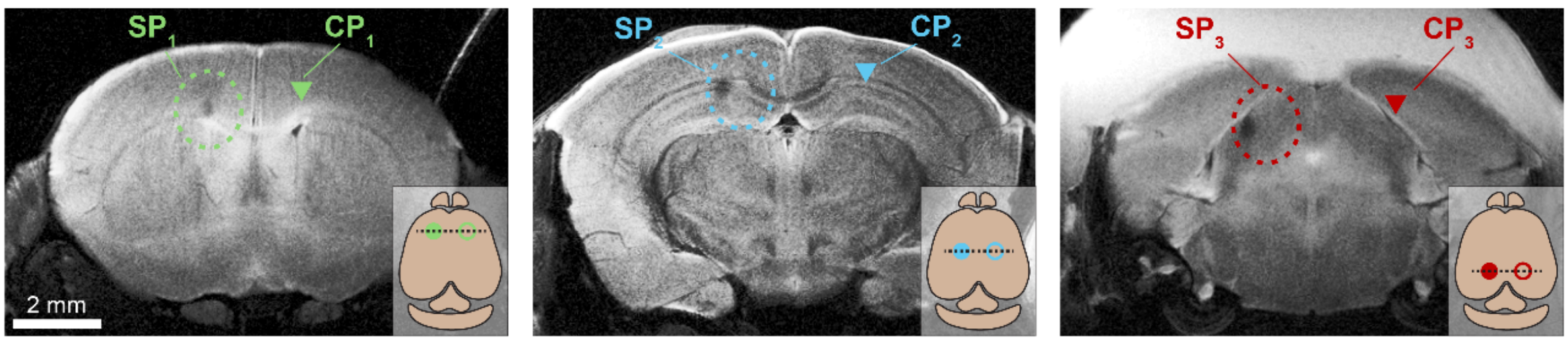

- We have expanded section “**Methods: MRI scanning and image processing**” with the corresponding acquisition parameters, related to the new set of MR images:
  “In the mouse model, all MRI images for post-FUS validation were acquired by a horizontal bore small animal 7T MRI scanner (PharmaScan, Bruker BioSpin, Germany) using a CryoProbe with the software ParaVision 6.0.1. Adjustments to the basic frequency, reference power, shimming were performed before the data acquisition. We applied a high resolution 2D RARE sequence to acquire 16 sagittal slices, with the following parameters: Repetition Time = 2500 ms, Echo Time = 11.2337 ms, FOV = 16 mm × 16 mm, Matrix Size = 267 × 267, Resolution = 60 µm × 60 µm, Slice Thickness = 0.4 mm, Slice Gap = 0 mm; RARE Factor = 6, Averages = 8, Acquisition Time = 14 min 40 s. Subsequently, we used a 2D RARE sequence to acquire 20 coronal slices with high resolution using the following parameters: repetition time of 4000 ms, echo time of 59.84 ms, echo spacing of 19.946 ms, bandwidth of 32894.7 Hz, FOV of 10 mm by 12.7 mm, matrix size of 400

× 512, resolution of 25 µm by 25 µm, slice thickness of 0.4 mm, slice gap of 0.1 mm, RARE factor of 8, and average of 16.”

- Since repeating the ex vivo mouse experiments have led to the same results, this reinforces the reproducibility of the lesions, as well as an improved and consistent image quality. To emphasize this aspect, we have included the following in **Discussion**:
  “The strength of this reproducibility is further supported by MRI scans from multiple specimens, each sonicated in the three regions along the anterior-posterior axis, all of which showed consistent lesion size and depth.”

- MR images originally presented in Fig. 4 have been post-processed using ImageJ, by applying brightness/contrast adjustments. In the revised manuscript, this figure is listed as **Supplementary Fig. 2**:

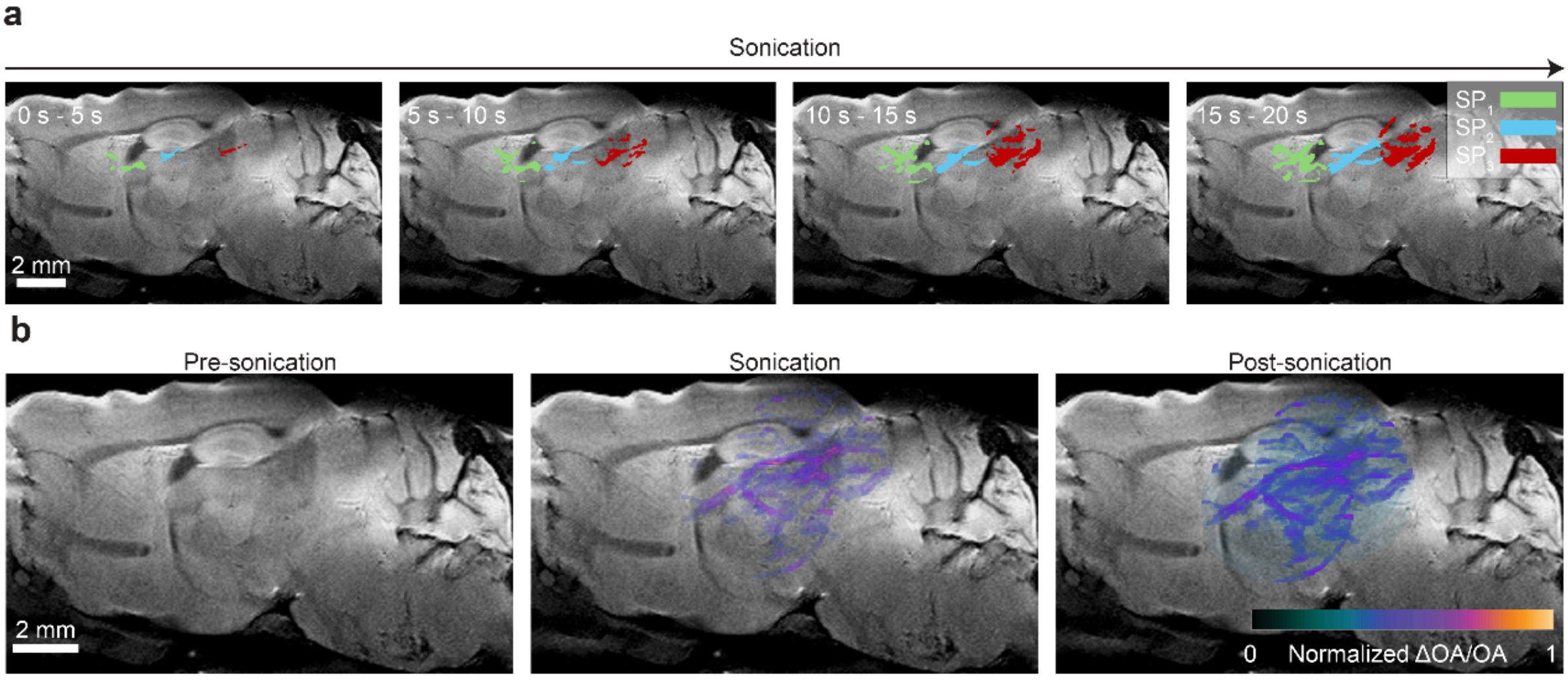

(1) Is the OA signal quantitative with temperature or do authors think that they can be quantitative? If not, then it would be better to rewrite the introduction to clarify that while MRI is more expensive, it offers quantitative thermometry.

Reply: The Reviewer is correct that MRI provides quantitative thermometry. Nevertheless, the low acquisition rate of MRI doesn’t make it suitable for on-the-fly live tracking of tissue changes. Our work, however, intentionally focuses on detecting the irreversible thermal damage directly, rather than on estimating the absolute temperature. This is because the relationship between OA signal and temperature is highly dependent on tissue properties, which vary between specimens and across different brain regions. Relying on a calibrated temperature threshold could therefore lead to inaccuracies and unsuccessful treatments, not leading to the desired thermal coagulation. By instead of using the OA signal’s intrinsic changes to identify the onset of coagulation, we created a quantitative method that is more robust to biological variability.

We have done the following modifications in the manuscript to tackle this question:

- We have explicitly stated that MR thermometry is quantitative in the **Introduction**, as well as highlighted its high cost as a counterpart:
  “Magnetic resonance image guided high-intensity focused ultrasound (MRgHIFU) combines magnetic resonance imaging (MRI) with quantitative MR thermometry, enabling highly sensitive detection of tissue temperature changes and real-time

monitoring of HIFU ablation. However, its high cost, the need for MRI-compatible materials, and prolonged procedure times limit its widespread clinical use."

- In the **Discussion**, we have emphasized the use of ΔOA/OA as an indicator of coagulation, independent of temperature measures:
  "Notably, ΔOA/OA provided a reliable real-time biomarker for thermal lesion formation, without relying on temperature measurement, as independently validated by MRI and histological analysis."

- We have revised the **Discussion** to state more clearly that our method aims to provide a quantitative and direct assessment of lesion formation without relying on thermometry:
  "Our approach enables brain vasculature imaging, FUS delivery, and a quantitative, assessment of thermal lesion formation in the tissue around the focus, that does not rely on temperature estimation."

(2) How you get to an imaging resolution of 780 nm needs some explanation. In addition, there are no OA images presented. I would like to see this 780 nm resolution. How is all the data handled? Across a mouse brain of 10 mm x 5 mm x 5 mm, this is 10^12 pixels. Presumably, the imaging is done over a much smaller FOV. But all this needs clarity.

Reply: Regarding the lack of OA images presented in the paper, we have not considered this initially because several other works from our lab, using the same system, have already published such data.[2–4] However, we agree that providing OA images will make the work more complete. In order to answer the concerns raised in this question, we have:

- Prepared a new figure, provided as supplementary material (**Supplementary Fig. 1**):

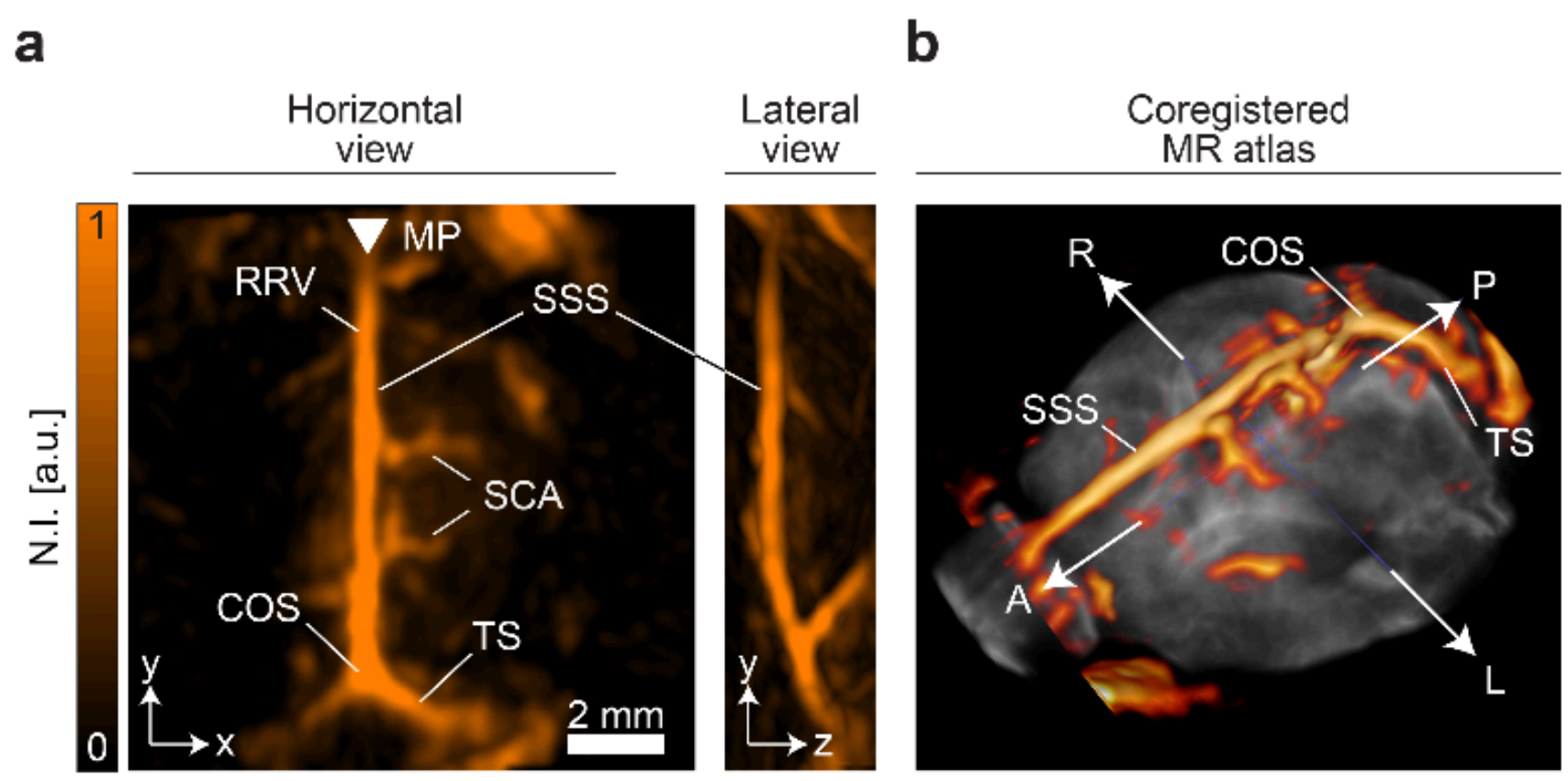

- We have included references in the main text to this new figure aiming to provide the reader with a wider context on the OA imaging aspect of the paper.

- We have specified the image resolution of the OA images in "**Methods: Optoacoustic guided HIFU system**":
  "OA images were acquired over a field of view ranging from 10 × 10 × 10 mm^3 to 12 x 12 x 12 mm^3 using a 128 × 128 × 128 voxels grid, corresponding to spatial sampling of approximately 78 μm/voxel to 93 μm/voxel, respectively."

- The magnitude 780 nm shows the excitation wavelength of the optical source and does not represent resolution; instead the excitation wavelength is selected based on the target tissue's optical absorption spectrum. For example, oxygenated and deoxygenated hemoglobin exhibit prominent absorption peaks near 850 nm and 750 nm respectively. On the other hand, **Fig. 1a** incorporates this aspect in the schematics of the setup, thus indicating that the laser is operating at this wavelength. Additionally, we have specified in **"Results: OA guided FUS system**" that the magnitude *780 nm* is referring to the wavelength the laser is operating with for producing the OA images:
  "The OAgFUS system (**Fig. 1a**) enabled real-time optoacoustic (OA) imaging with a laser operating at a wavelength of 780 nm for anatomical navigation and target localization in the ex vivo mouse brain (**Supplementary Fig. 1a**), following skull removal and sample positioning."

(3) It is not clear in Fig. 3 Why the inner VOI doesn't have as high a signal change as the outer VOIs. This needs explanation. To first order, no matter where the VOI is spatially, coagulation will occur at some temperature, the OA signal is the same as a function of temperature, so the coagulation should be at the same OA level. It makes no sense that the OA signal is higher in the outer VOIs. Maybe there is something about the way these signals are normalized, but this needs clarity.

Reply: We agree that if the OA signal were a universal function of temperature, the observed pattern would indeed be counterintuitive. We hypothesize this observation, relative to the plots of SP_1 in **Fig. 3c**, is resolved by considering the dynamic optical transformation induced by thermal coagulation and the heterogeneity of brain tissue.
As the coagulative region emerges at the focus and expands within the inner VOI, its optical scattering coefficient increases irreversibly. This severely attenuates the local optical fluence available for OA conversion, causing the signal within the core to plateau or decline despite elevated temperature. On the other hand, the signal in the outer VOI might be enhanced due to both temperature increase (thermal diffusion) and the light scattered from the inner, already coagulated tissue. The combined effect produces a more rapid rise in OA signal amplitude in the outer VOI than in the inner VOI.
We have made the following updates in the manuscript to provide clarity on this question, and added new bibliography:

- We have extended the **Discussion** to directly address the Reviewer's question:
  We observed a distinctive ΔOA/OA profile in some of the traces (e.g., SP_1, **Fig. 3C**) where the OA signal increase in the outer VOIs surpassed that of VOI_1. This pattern, which would be paradoxical if OA signal were a simple function of temperature, is consistent with a hypothesis of dynamic optical property change during coagulation. As the focal core coagulates, its optical scattering increases irreversibly. This depletes the local optical fluence available for OA conversion. Simultaneously, light scattered from this opaque region may enhance the fluence (and thus the OA signal) in the surrounding heated tissue. Consequently, the spatial peak of the OA signal can transiently mark the advancing coagulation boundary rather than the temperature maximum. We posit that this phenomenon is mediated by local optical heterogeneity and the specific spatiotemporal progression of coagulation, explaining why it is prominently observed only in a subset of the acquired datasets.

- We have extended "**Methods: OA signal analysis**" to better explain the whole extent of the signal analysis procedure we have followed:

“After computing the relative change in OA with respect to the baseline (ΔOA/OA) for every voxel, we discarded those voxels that exhibited a decrease in this value during the first few seconds into sonication. Since such decay is not physically plausible, we attributed it to artifacts arising from the partial angular coverage of the transducer.”

(4) The Figure 3 T2s are motion corrupted. It’s really hard to see the lesion with this level of artifact.

Reply: The artifacts seen in the previous version of Fig. 3 were likely not due to motion, since the experiments were done ex vivo, but were probably related to brain deformation due to the physical pressure of the coupling water in the transducer and susceptibility artifacts at the brain edges during MRI acquisition. We have made the following updates in the manuscript, that allowed us to prevent such deformation:

- We have explained in “**Methods: Rodent preparation and integration in the setup**” the key point that avoids the deformation of the brain, that was clearly visible in Fig. 3f of the original manuscript:
  “We gently moved the mouse brain toward the foil window, taking care not to press it against the surface.”

Additional changes made during revision:

- We updated the quantitative analysis underlying **Fig. 4e** (refered to as Fig. 5e in the initial submission) after refining the data processing workflow during revision. This resulted in minor numerical adjustments to the plotted values. The updated figure and corresponding values in the text have been revised accordingly. These changes do not affect the interpretation or conclusions of the study.

1. Kim, J. *et al.* Real-Time Photoacoustic Thermometry Combined With Clinical Ultrasound Imaging and High-Intensity Focused Ultrasound. *IEEE Trans. Biomed. Eng.* **66**, 3330–3338 (2019).
2. Ni, R. *et al.* Multiscale optical and optoacoustic imaging of amyloid-β deposits in mice. *Nat. Biomed. Eng.* **6**, 1031–1044 (2022).
3. Estrada, H. *et al.* High-resolution fluorescence-guided transcranial ultrasound mapping in the live mouse brain. *Sci. Adv.* **7**, eabi5464 (2021).
4. Deán-Ben, X. L. *et al.* Deep optoacoustic localization microangiography of ischemic stroke in mice. *Nat. Commun.* **14**, 3584 (2023).

Point to point response to the Reviewer's comments

Reviewer #1 (Remarks to the Author):

This manuscript has been thoroughly revised in response to the reviewers comments, resulting in an improvement in quality that meets the standards of Nature Communications. In particular, the objections regarding clinical relevance and novelty were addressed by conducting additional experiments as part of an in vivo animal study on sheep. In addition, new MR images were taken with better image quality, from which the clinically relevant information is now clearly visible.

In general, the presented work on Optoacoustic guided high intensity focused ultrasound is of great interest for the biomedical research community in terms of diagnosis and treatment. Furthermore, the work has been carried out in a technically sound manner, and the data supports the conclusions. Which brings me to the decision to recommend the work for publication in Nature Communication.

Reply: We thank the reviewer for the positive reassessment of our revised manuscript and for recognizing the added in vivo sheep study and improved MR imaging.

Reviewer #2 (Remarks to the Author):

The "Integrated device for optoacoustically-guided ultrasound surgery" proposed an interesting approach of integrating real-time optoacoustic tomography with concurrent focused ultrasound delivery using a single high-frequency spherical transducer array. Developing such an integrated system and demonstrating its feasibility in both mouse and sheep models is highly significant for the field of image-guided therapy. While the potential of the fusion system is impressive, I believe that major revisions are required to ensure the manuscript has the potential to meet the high standards of the journal.

Reply: We thank the Reviewer for recognizing the significance and clinical potential of the integrated optoacoustically guided ultrasound surgery platform. We have thoroughly revised the manuscript to strengthen its technical clarity, translational significance, and overall presentation, including clarifying the novelty of the work, expanding the discussion of advantages and limitations, and reinforcing the in vivo and ex vivo validation results. We hope that these revisions satisfactorily address the Reviewer's comments.

1. Specifically, the advantages of the OAgFUS system should be clearly demonstrated through two main aspects: (1) OA structural-based target navigation during FUS and (2) simultaneous OA-based lesion monitoring. The current presentation of the sheep data in Figure 5 could be further strengthened to fully support the "OA-guided" aspect of the OAgFUS system. Although the authors mention that OA imaging offered valuable structural information to avoid vasculature during the procedure, the manuscript would benefit from the inclusion of representative OA images of the sheep brain, similar to those provided for the mouse model. Additionally, the current results (in sheep model) seem to have some limitations in fully demonstrating the significant clinical potential of the OAgFUS system. Given that the sheep data appears to be limited to a single specimen, merely showing the changes in OA signals from VOI1 to VOI3 does not sufficiently showcase this potential and

further direction. I suggest that the authors consider adding further analysis based on this result, such as a more detailed assessment of the lesion dimensions in comparison with the histological findings. Overall, I suggest supplementing the results to more clearly demonstrate the significant advantages inherent to the OAgFUS system.

Reply: Regarding the representative OA images of the sheep brain:

- We have complemented **Supplementary Fig. 1** with a new panel **c** by including representative OA images of the sheep brain, thereby better illustrating the OA-based structural guidance achieved in the large-animal experiment.

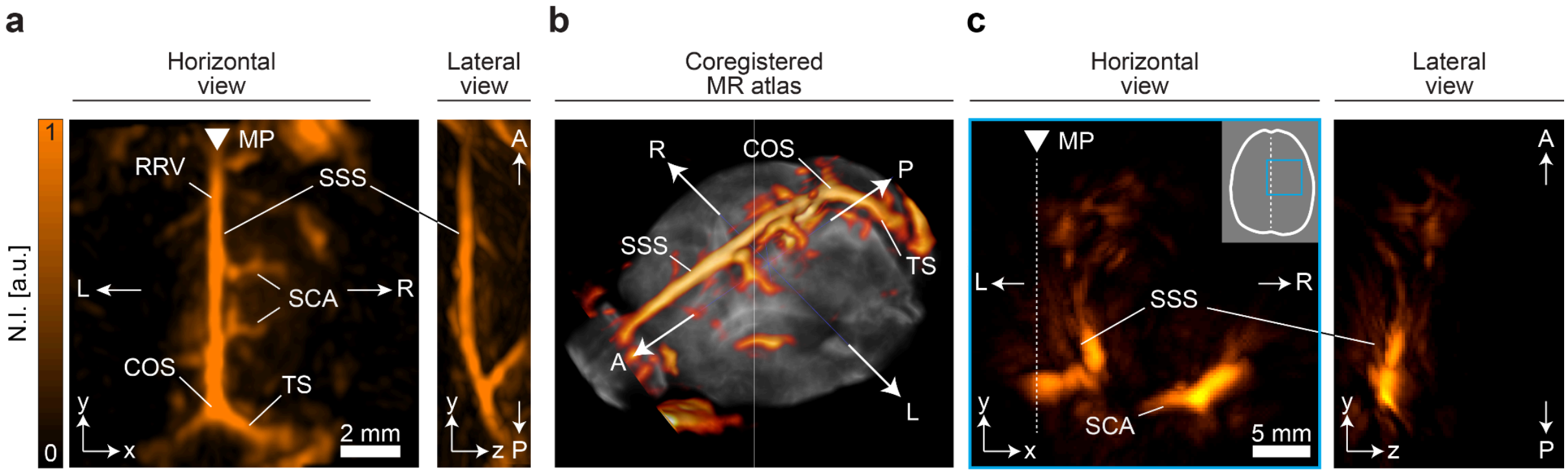

"**Supplementary Figure 1. OA imaging at 780 nm reveals blood vessels and vascular structures in the mouse and sheep brain**. **a**. OA image reconstruction representing a single time point from the pre-sonication phase (**Fig. 1b**) of the experiments, acquired using a laser source wavelength of 780 nm. The different windows show the maximum intensity projections (MIPs) aligned with the horizontal and sagittal views. The white arrow indicates the position and direction of the midplane (MP). The imaged FOV is 12 × 12 × 12 mm^3. **b**. 3D reconstruction of the vascular network from **a**, registered to an MR atlas. **c**. Volumetric OA MIPs of the sheep brain (FOV is 30 × 30 × 20 mm^3). The white arrow indicates the position and direction of the MP. Superior sagittal sinus (SSS); Transverse sinus (TS); Superior cerebellar arteries (SCA); Confluence of sinuses (COS); Rostral rhinal veins (RRV); Left (L); Right (R); Anterior (A); Posterior (P)."

- We revised "**Results: In vivo validation in a large animal model"** section to include the appropriate reference to the newly added supplementary panel, ensuring consistency between the description of the sheep imaging procedure and the corresponding OA image provided in Supplementary Fig. 1c.:
  "The OAgHIFU system further enabled real-time OA imaging with a laser operating at a wavelength of 780 nm for anatomical navigation and target localization in the sheep brain (**Supplementary Fig. 1c**)."

Regarding the limitations of the results in the sheep model, we refined the OA-based lesion analysis by increasing the spatial sampling of the concentric VOIs to improve the consistency and spatial resolution of the analysis, thereby providing a more informative estimate of the spatial extent of the lesion. Importantly, this updated analysis does not change the overall interpretation of the data, but strengthens the OA-based evidence for simultaneous lesion monitoring in the sheep experiment. To address this point, we have:

- Updated **Fig. 5b** according to the refined lesion analysis:

[REDACTED]

- Revised the text in "**Results: In vivo validation in a large animal model**" to reflect this updated VOI definition:

"We defined a SP 1 cm below the cortical surface (**Fig. 5a**, right), located in the mesial temporal-diencephalic junction, involving adjacent hippocampal, thalamic/hypothalamic, and piriform lobe structures. We computed the mean ΔOA/OA for the different VOIs, using larger values of R_1 and R_2 to account for the larger focus (**Fig. 5b**, **Fig. 3b,** and **Methods**). In the resulting traces, ΔOA/OA for all VOIs starts to increase due to the temperature rise from the FUS-mediated thermal energy deposition. Approximately 9 s into sonication, VOI_1 undergoes a marked trend change, followed by VOI_2 approximately 3 s after. VOI_3 and VOI_4 show minor corrections. These results indicate that the lesion-associated OA response is clearly captured within VOI_2. Since the in vivo traces exhibited motion-related and physiological fluctuations, we refined the lesion size , by examining the temporal derivative of ΔOA/OA during sonication (**Methods** and **Supplementary Fig. 4**). This refined analysis localized the lesion boundary within VOI_3, corresponding to an estimated lesion width of 1-1.5 mm.

After HIFU, we performed a postoperative MR (**Fig. 5c**, **Methods**). The assessment of T2-weighted MRI reveals a clearly distinguishable area in the target SP, characterized by a hyperintense area with central hypointensity. The lesioned area was evaluated in formalin-fixed paraffin-embedded brain tissue sections. In the H&E-stained sections, rarefication of the cerebral parenchyma was observed in the white matter of the cerebrum. In addition to tissue ruptures, small accumulations of pale eosinophilic fluid were present. Increased cellularity in terms of gliosis or inflammatory cell infiltration was not detected (**Fig. 5d**, **Methods**). In the corresponding histological section, the lesion measured approximately 1.6 mm in width, which is close to the refined OA-based estimate range, noting that the

histological assessment was based on a single 2D section and therefore provides a partial representation of the full 3D lesion extent."

- Revised "**Methods: Quantitative analysis of lesion dimension**" to describe the quantitative approach used:
"For the in vivo sheep experiment, the OA signal analysis shown in **Fig. 5b** was performed using concentric ellipsoidal VOIs spaced by 250 μm in the radial direction. For each VOI, we extracted the minimum derivative value during sonication and used a negative derivative threshold to identify the abrupt OA downturn associated with coagulation. The outermost splitted VOI satisfying this criterion was then used to define the refined lesion-width estimate. In the present study, the derivative threshold was set to -5 %/s, which derives into a lesion size of 1-1.5 mm wide."

- We have added a new figure (**Supplementary Fig. 4**) to illustrate the temporal derivatives of ΔOA/OA during sonication in the in vivo sheep experiment. This figure supports the refined lesion-size analysis by showing the derivative-based criterion used to localize the lesion boundary within the lesion-associated region identified in **Fig. 5b**.

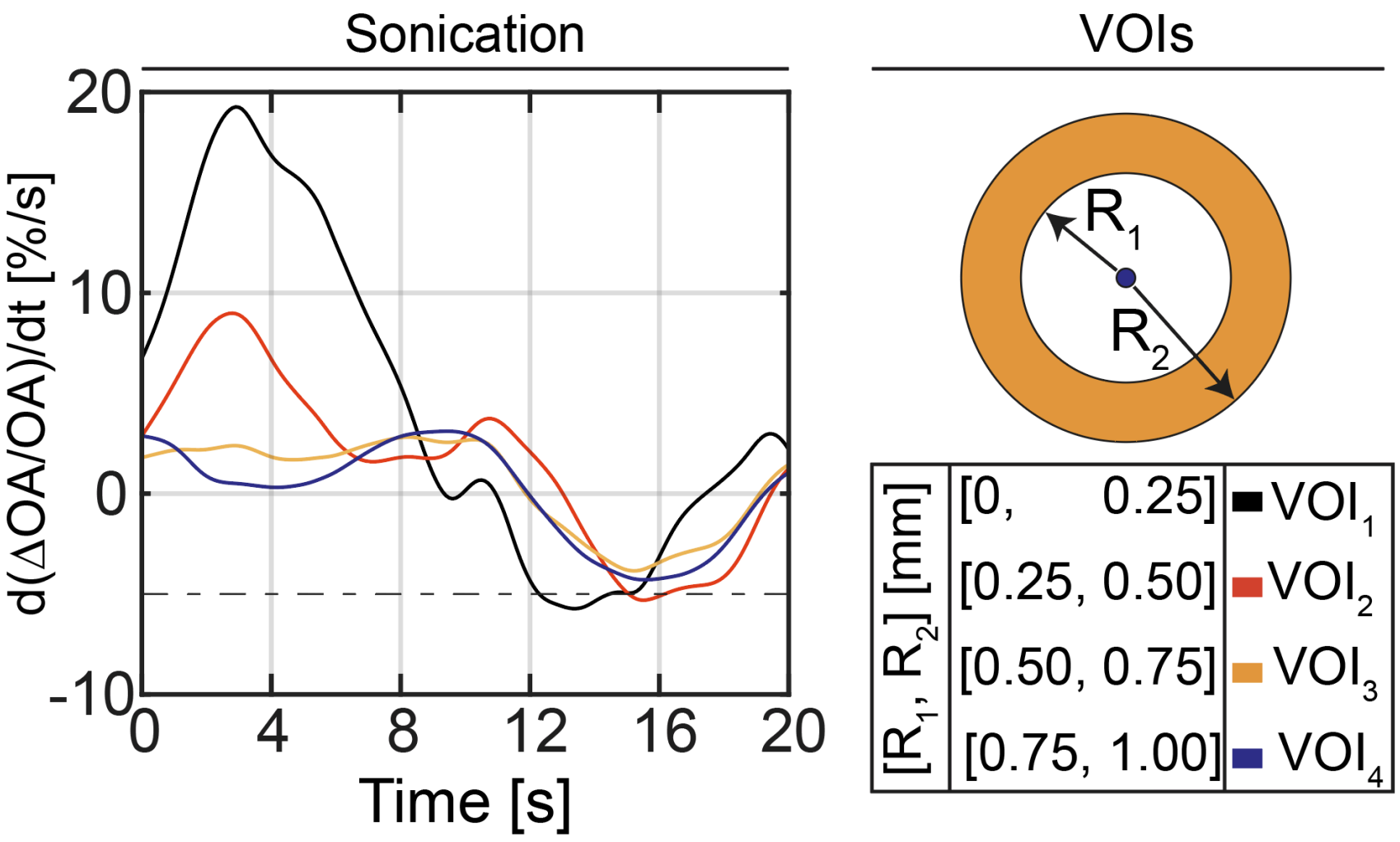

Supplementary Figure 4. Temporal derivatives of ΔOA/OA during sonication in the in vivo sheep experiment. Temporal derivatives of the relative OA signal change (ΔOA/OA) for the concentric ellipsoidal VOIs analyzed in the in vivo sheep experiment, shown over the sonication time window only. The curves were used to refine lesion-size estimation within the lesion-associated region identified in **Fig. 5b**. The horizontal dashed line indicates the derivative threshold used to identify the abrupt signal downturn associated with coagulation of -5 %/s.

2. There is a discrepancy in figure referencing within the Methods section (in ‘Quantitative analysis of lesion dimension’ section). The authors refer to "Fig. 3b bottom row" as showing the OA signal's exponential increase for "mere heating". But it seems that there are missing. Additionally, the authors stated that a high R2 from exponential fitting was assumed to indicate a thermal lesion, but wouldn't a high R2 actually be indicative of "mere heating" instead? Please check this point.

Reply: We have corrected the discrepancies in the references to Fig. 3. In the reviewed manuscript, “Fig. 3c top row” now refers to the traces relative to the sonication points, whereas “Fig. 3c bottom row” refers to the traces corresponding to the control points. Regarding the R2 coefficient, we corrected the text accordingly: high R2 from the exponential fitting is indicative of “mere heating”, not a thermal lesion.

3. The current methodology for predicting lesion size based on OA signals relies on several assumptions, making it difficult to clearly interpret the accuracy of the results, while authors claim that the OA-derived VLrange closely approximates the histological findings. However, this claim appears somewhat vague, as it is based on the average histological volume falling within the relatively wide, predefined ranges of VOI2 or VOI3. To provide a more persuasive validation, the authors should consider to examine the feasibility of providing a one-to-one correspondence between the OA-estimated range and the individual histological measurement for each of the seven lesions. Additionally, the authors should consider including a representative figure that displays the specific OA signal traces alongside the corresponding histological lesion size for a single case to better illustrate the correlation between the two modalities. Overall, please consider providing sufficient justification to demonstrate the rigor of the methodology used for estimating lesion size.

Reply: We performed a lesion-by-lesion comparison between the OA-based VOI assignment and the corresponding histology-derived lesion volume for all seven lesions, and added this analysis as **Supplementary Fig. 3**.
This updated comparison shows direct agreement in 4 of the 7 lesions, in which the histology-derived volume falls within the OA-estimated lesion-volume range. In the remaining 3 lesions, the OA-based estimate falls in the adjacent lower volume range rather than the corresponding histology-derived range. Importantly, these mismatches are limited in magnitude and remain close to the boundary between the two ranges, indicating that the discrepancy is not large in absolute volumetric terms. Thus, even in the non-matching cases, the OA-based framework preserves lesion-size ordering and provides a close approximation of lesion extent at the level of coarse range classification.
We further note that the purpose of the present method is not exact volumetric segmentation, but rather real-time intra-procedural estimation of lesion extent from OA signals. In that context, the observed behavior is meaningful for two reasons. First, the agreement in more than half of the lesions supports the feasibility of the proposed range-based framework. Second, in the remaining cases, the OA-based estimate tends to assign the lesion to a neighboring and generally smaller range, indicating conservative behavior rather than substantial overestimation of tissue damage.
We have revised the manuscript to explicitly include the new lesion-by-lesion comparison. More specifically:

- We have revised “**Results: OA monitoring of HIFU-induced lesions**” accordingly: “Next, we used the OA signals to estimate dimensions of the induced lesions (**Methods**). Specifically, we identified the outermost VOI (OM-VOI) that encapsulates the lesion, and extracted the lesion size range ($V_{L,range}$, **Fig. 4e**). Based on this analysis, 3 out of 7 SPs were assigned to the $V_{L,range}$ corresponding to VOI_2, including

SP_2 and SP_3 (**Fig. 4e**, red range plot) whereas the remaining 4 to the $V_{L,range}$ corresponding to VOI_3, including SP_3 (**Fig. 4f**, orange range plot). To evaluate these estimates, we calculated the lesion volume (V_L) in the H&E-stained sections as ground truth, using the enhanced-contrast images (**Fig. 4d**), obtaining an average lesion size $V_{L,avrg}$ = 1.72 mm^3 (n=7, **Fig. 4e** and **Fig. 4f**). In addition, to assess the correspondence between the OA-based range estimate and histology on a lesion-by-lesion basis, we compared the OA-estimated volume range with the histology-derived lesion volume for each of the seven lesions (**Supplementary Fig. 3**). This comparison showed direct agreement in 4 of the 7 lesions. In the remaining 3 lesions, the OA-based estimate fell in the adjacent lower volume range while remaining close to the corresponding histology-derived volume, indicating conservative underestimation rather than substantial discrepancy. Using the midpoint of each OA-estimated lesion-volume range as a simple summary statistic, the comparison with histology yielded a mean discrepancy of −0.17 mm^3 and a mean absolute discrepancy of 0.73 mm^3, indicating limited overall bias and moderate lesion-by-lesion deviation (**Methods**). Together, these results support the use of the OA-based framework as a bounded, range-based estimate of lesion size rather than precise volumetric quantification."

- We have revised: "**Methods: Quantitative analysis of lesion dimension**" to describe the metric used to quantify the discrepancy between the OA-based lesion estimates and the histology-derived lesion volumes:
  "To summarize the discrepancy between $V_{L,range}$ and V_L, we used the midpoint of each $V_{L,range}$ range as a simple scalar summary statistic. For each SP, this midpoint was calculated as the average of the lower and upper bounds of the corresponding $V_{L,range}$, and the discrepancy was defined as the midpoint-based $V_{L,range}$ estimate minus V_L. We then calculated the mean discrepancy and the mean absolute discrepancy across all SPs. Because the OA framework provides a bounded range rather than a single point estimate, this midpoint-based discrepancy was used only as an approximate descriptive metric."

- We have also revised the **Discussion** to better clarify the scope, limitations and interpretation of the range-based lesion-size estimation approach:
  "To enable practical lesion size estimation in real time, we introduced a range-based approach ($V_{L,range}$) derived from OA signals. This method allowed us to consistently estimate the lesion extent across SPs. To validate this approach, we compared the OA-based estimates with histological measurements obtained from contrast-enhanced H&E sections, which yielded an average lesion volume of $V_{L,avrg}$ = 1.72 mm^3. A lesion-by-lesion comparison further showed that the OA-based framework provides a bounded estimate of lesion extent in most cases, while the remaining mismatches reflected conservative underestimation of lesion size (**Supplementary Fig. 3**). Consistent with this interpretation, midpoint-based comparison yielded a mean discrepancy of −0.17 mm^3 and a mean absolute discrepancy of 0.73 mm^3, indicating limited overall bias despite the deliberately coarse range-based formulation.
  In the in vivo sheep experiment, a refined derivative-based analysis yielded an estimated lesion width of 1–1.5 mm, which aligns closely with the histological measurement of approximately 1.6 mm (**Fig. 5d**). These findings validate the

method's efficacy for real-time monitoring while simultaneously highlighting its limitations in achieving exact volumetric delineation."

- We have added a new figure (**Supplementary Fig. 3**) to provide the lesion-by-lesion one-to-one comparison between the OA-estimated volume range and the corresponding histology-derived lesion volume:

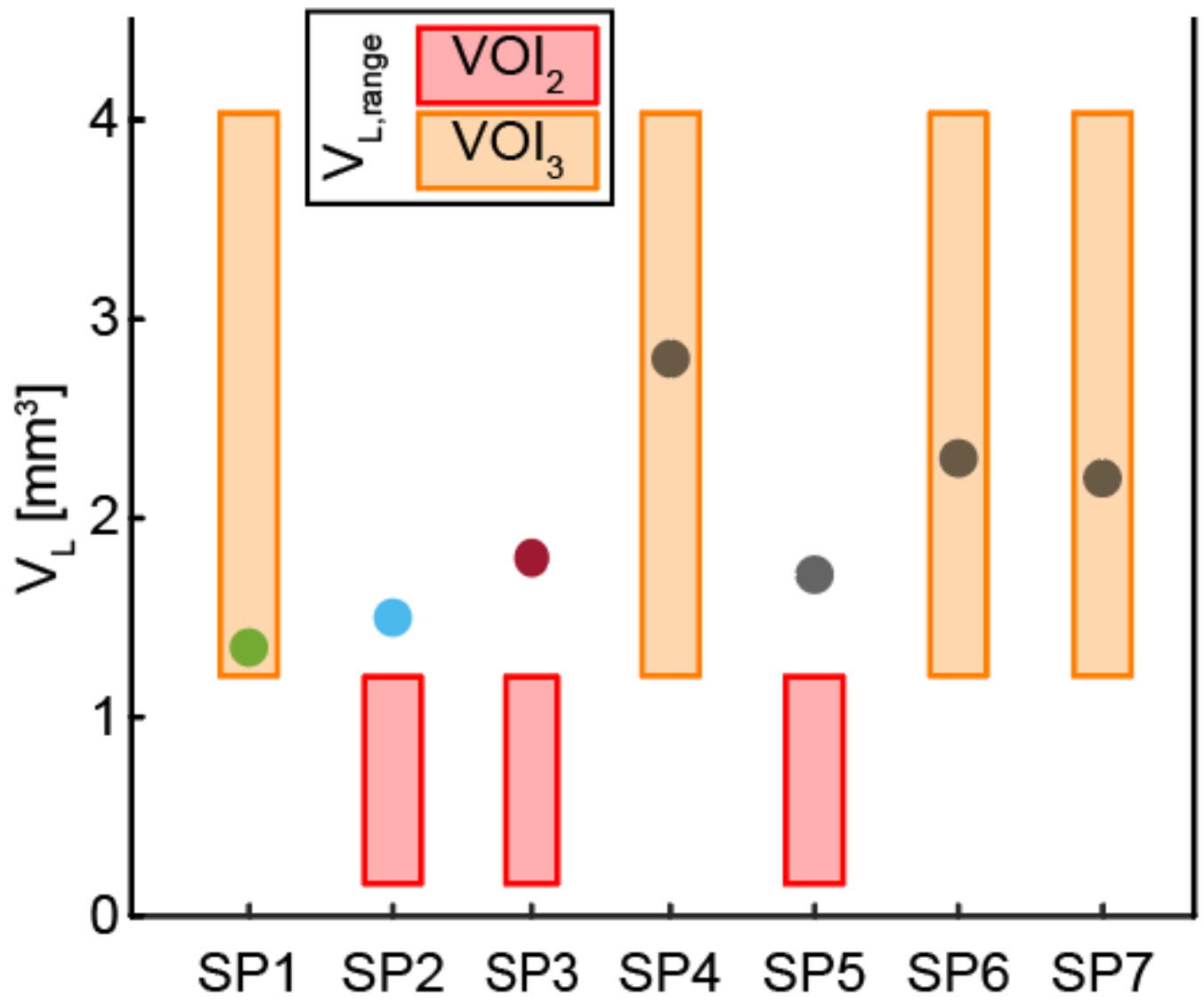

Supplementary Figure 3. Lesion-by-lesion comparison between OA-based lesion-volume range estimation and histology-derived lesion volume. For each SP, colored bars denote the lesion-volume range estimated from the OA analysis ($V_{L,range}$, **Fig. 4e**), and symbols denote the corresponding lesion volume quantified from H&E-stained histological sections (V_L, **Fig. 4d**).

4. The current estimation of lesion volume appears to be conducted within a very broad range. Please providing a more detailed discussion on the clinical significance of this approach in the aspect of further directions, along with strategies or directions for achieving more precise lesion evaluation, would help convince readers of the potential demonstrated in this study.

Reply: We have expanded the **Discussion** to clarify the clinical significance of this strategy: "In this context, the current lesion-evaluation framework should be interpreted as an empirical range-based method for intra-procedural assessment. Our implementation infers the lesion extent from discrete concentric ellipsoidal VOIs centered on the acoustic focus, such that the resulting estimate is bounded by the shell spacing used in the analysis. Its precision is further influenced by the spatial resolution of the OA system, the finite voxel size, the elongated geometry of the HIFU focus, and tissue-dependent thermal and optical heterogeneity. While comparisons with histology are further complicated by uncertainties in tissue sectioning and co-registration, the approach remains highly relevant; it provides critical real-time feedback by detecting lesion onset and ensuring the ablated region remains within the intended treatment zone. Future work should focus on improving lesion delineation through continuous 3D boundary estimation from the OA signal distribution, quantification strategies that explicitly account for the system point spread function, or combining OA monitoring with complementary imaging modalities such as pulse-echo ultrasound for improved boundary definition and validation."

In our view, the main translational value of the current method lies in providing immediate feedback during sonication, enabling the detection of lesion onset, and estimating whether the treated region remains confined to the intended focal zone. For clinical use, such real-time bounded feedback may already be valuable even before exact volumetric quantification is achieved.

5. What does the standard deviation (shaded area) in Fig 3d stand for? Please clarify this.

Reply: The shaded area represents the standard deviation calculated across all the voxels within VOI1, whose average signal is shown as the mean trace (solid line). We have clarified this in the revised caption of **Fig. 3d** by providing a more detailed explanation of its contents: "**Figure 3**. **d**. Mean ΔOA/OA trace corresponding to VOI_1 (solid line), calculated as the average signal across all voxels within VOI1; the shaded region indicates the standard deviation across voxels."

6. In Fig 5b, please also display the VOI information to ensure consistency and clarity.

Reply: We agree that indicating the VOI information in **Fig. 5b** will increase consistency and clarity. In the revised version, we have updated Fig. 5, as shown in one of the previous replies.

Regarding **Fig. 5d**, we have updated the histology panel due to an error in the alignment of the multiple histological sections with the MR images. After carefully reviewing the co-registration and section matching, we replaced the incorrect histological image with the corresponding section and revised the figure accordingly.

Reviewer #3 (Remarks to the Author):

The manuscript is much improved.

Thank you for the clarification that 780 nm is the laser wavelength. It would be helpful to specify this in the caption to Fig. S1.

Fig. S2. is somewhat incomprehensible and would benefit from clarification.

Reply: We thank the Reviewer for this positive assessment and for noting that the manuscript is much improved after revision.

Regarding the wavelength, we have revised the caption of Fig. S1 and specified this aspect: "**Supplementary Figure 1**. **OA imaging at 780 nm reveals blood vessels and vascular structures in the mouse and sheep brain**. **a**. OA image reconstruction representing a single time point from the pre-sonication phase (**Fig. 1b**) of the experiments, acquired using a laser source wavelength of 780 nm. The different windows show the maximum intensity projections (MIPs) aligned with the horizontal and sagittal views. The white arrow indicates the position and direction of the midplane (MP). The imaged FOV is 12 × 12 × 12 mm^3. **c**. Volumetric OA MIPs of the sheep brain (FOV is 30 × 30 × 20 mm^3). The white arrow indicates the position and direction of the MP. Superior sagittal sinus (SSS); Transverse sinus (TS); Superior cerebellar arteries (SCA); Confluence of sinuses (COS); Rostral rhinal veins (RRV); Left (L); Right (R); Anterior (A); Posterior (P)."

Regarding **Supplementary Fig. S2**, we agree that the figure was not sufficiently clear in the previous version. In response, we have:

- Revised the corresponding main-text description to clarify the content of each panel: "To better visualize the FUS-induced tissue response, we registered the 3D volumetric OA data to the T2-weighted MRI images (**Supplementary Fig. 1b** and **Methods**). This co-registration enabled anatomical visualization of ΔOA/OA at the sonication sites over time (**Supplementary Fig. 2a**), as well as a phase-wise comparison of the normalized ΔOA/OA response across all the SPs (**Supplementary Fig. 2b** and **Supplementary Movie 1**)."

- Revised the panel annotations to clarify the content of each panel:

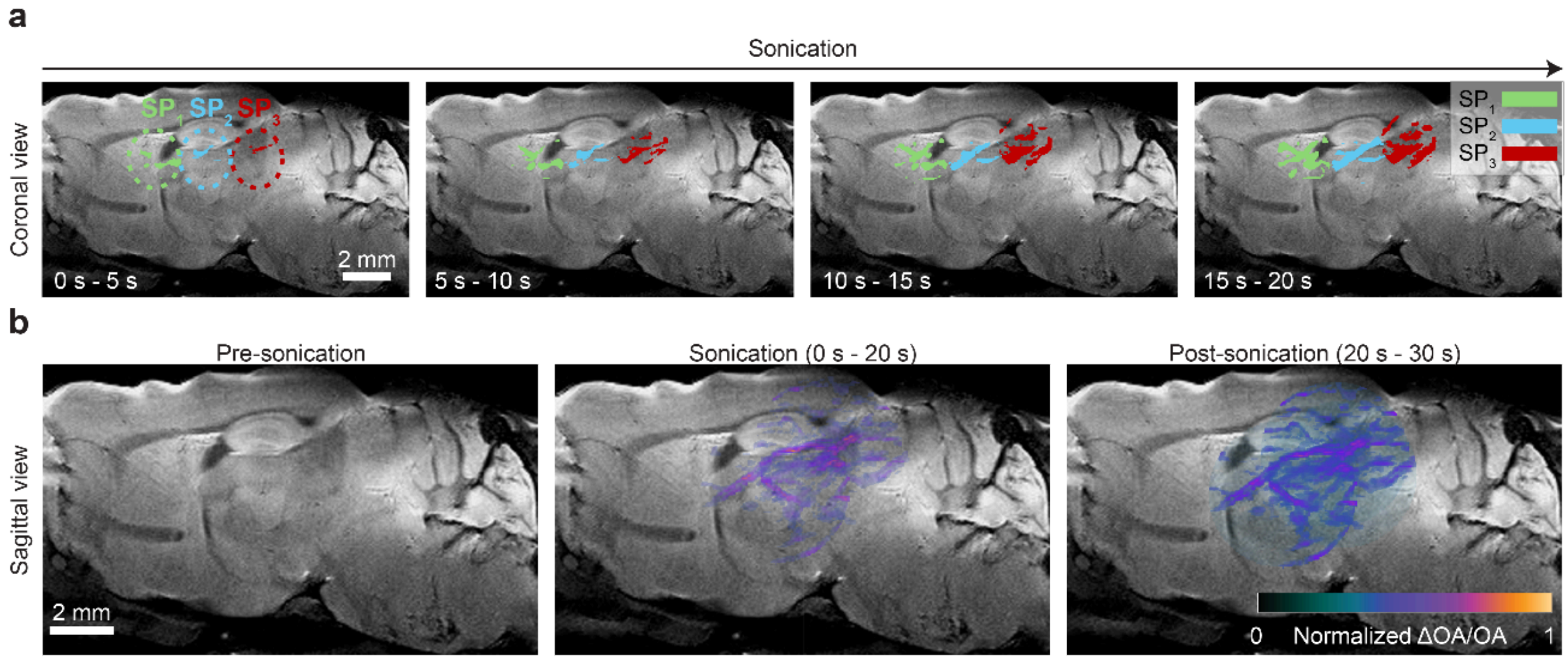

- Revised the figure legend. Specifically, we now explain more explicitly that panel **a** shows the temporal evolution of the MRI-registered relative optoacoustic signal change (ΔOA/OA) at representative time points, whereas panel **b** shows the phase-wise normalized ΔOA/OA response for SP1, SP2, and SP3 across the pre-sonication, sonication, and post-sonication periods. These revisions improve the readability of the figure and clarify its purpose within the manuscript: "**Supplementary Figure 2**. **MRI-registered visualization of OA changes during HIFU sonication**. **a**. Relative optoacoustic signal change (ΔOA/OA) overlaid on T2-weighted MRI sections after OA–MRI co-registration. Representative time points during sonication illustrate the temporal evolution of the OA response around SP1, SP2 and SP3, each represented with distinct colors. For visualization, OA data were temporally high-pass filtered (6 %) to emphasize dynamic signal changes. **b**. Normalized maps of relative OA change for all three sonication experiments (SP_1, SP_2, and SP_3) during different phases: pre-sonication, sonication (0 s - 20 s), and post-sonication (20 s - 50 s). Each heat map shows the maximum OA intensity change observed within the corresponding time window. OA data are spatially and temporally normalized and processed with a 6% temporal high-pass filter."

Version 3

Reviewer #2 (Remarks to the Author):

The authors have adequately addressed the concerns raised in the previous review. The inclusion of the large-animal OA images and the 1:1 lesion comparison data has clarified the system's capabilities and the validity of the methodology. Additionally, the limitations of the proposed approach are now properly discussed in the manuscript. I have no further comments and recommend the manuscript for publication.

Reply: We thank Reviewer #2 for the positive assessment of the revised manuscript. We are pleased that the added large-animal OA images, lesion-by-lesion comparison data, and expanded discussion of limitations have satisfactorily addressed the reviewer’s concerns.
